# Recent Progress in Developing Monolithic Perovskite/Si Tandem Solar Cells

**DOI:** 10.3389/fchem.2020.603375

**Published:** 2020-12-22

**Authors:** Na Liu, Lina Wang, Fan Xu, Jiafeng Wu, Tinglu Song, Qi Chen

**Affiliations:** ^1^Experimental Center of Advanced Materials, School of Materials Science and Engineering, Beijing Institute of Technology, Beijing, China; ^2^Department of Materials Science and Engineering, McMaster University, Hamilton, ON, Canada; ^3^Beijing Institute of Technology Chongqing Innovation Center, Beijing Institute of Technology, Beijing, China

**Keywords:** perovskite, SI, tandem solar cells, top electrodes, recombination layer

## Abstract

Monolithic perovskite/Silicon tandem solar cells have reached a certified efficiency of 29. 1% in recent years. In this review, we discuss material design for monolithic perovskite/Si tandem solar cells, with the focus on the top-cell development to improve their performance. Firstly, we introduce different types of transparent electrodes with high transmittance and low sheet-resistance used in tandem solar cells. We then discuss the development of the wide-bandgap perovskite absorber for top-cells, especially the strategies to obtain the perovskite layers with good efficiency and stability. In addition, as a special functional layer in tandem solar cells, the recombination layers play an important role in device performance, wherein different configurations are summarized. Furthermore, tandem device cost analysis is discussed. This review summarizes the progress of monolithic perovskite/Silicon tandem solar cells in a pragmatic perspective, which may promote the commercialization of this technology.

## Introduction

Crystalline silicon (Si) based solar cells are the mainstream technology, occupying 90% of the photovoltaic (PV) market, and their production costs have reduced dramatically in the past decade. Si based solar cells have achieved an average panel power conversion efficiency (PCE) of around 20% for modules in mass production. And a record cell efficiency of 26.7% has been achieved, which is very close to the Shockley-Queisser (S-Q) limit of single-junction solar cells (Lee et al., 2012; Zhou et al., 2014; Dong et al., 2015). To achieve higher efficiency, it thus requires an alternative material, which also provides diversity to the PV market. In recent years, perovskite (PVSK) solar cells with low preparation cost and convenient processing have attracted wide attention (Green et al., 2014). The PCE of PVSK solar cells has evolved from the initial 3.8% to a now certified 25.5%, within only one decade (Laboratory, 2019).

The solar irradiation spectrum exhibits a broad energy distribution, while the semiconductor material could only absorb a portion of photons with an energy larger than the bandgap. The PCE of a single-junction solar cell cannot exceed the S-Q limit due to the below-bandgap absorption loss and the thermal-relaxation loss of hot charge-carriers (Malinkiewicz et al., 2013). Integrating the high-efficiency wide-bandgap top solar cells with low-bandgap bottom solar cells to form tandem solar cells has therefore been considered as a promising strategy to improve their PCE beyond the S-Q limit (Rong et al., 2018). The tandem configurations allow the high-energy photons to be absorbed in the top-cell, which generates high voltage to reduce the thermalization loss, and also allows the bottom-cell to absorb the transmitted low-energy photons, which leads to a broader harvesting of the solar spectrum. It has been reported that theoretical maximum efficiency of Si based tandem cells could be increased from 29 to 42.5% (Bremner et al., 2008).

Halide PVSK and Si have different bandgaps, and the bandgap of PVSK materials could achieve a bigger bandgap through composition engineering (Jesper Jacobsson et al., 2016). Therefore, the PVSK solar cells can be used as top cells and the Si cells as bottom cells, which add up to perovskite/Silicon (PVSK/Si) tandem solar cells. In 2015, Mailoa et al. first demonstrated a 1 cm^2^ 2-terminal (2T) monolithic PVSK/Si solar cells with a V_OC_ of 1.65 V and a PCE of 13.7%. During the past years, extensive studies have been conducted with the focus on photocurrent non-uniformities (Song et al., 2016), PVSK absorber optimization (Qiu et al., 2018; Chen B. et al., 2019; Yang et al., 2019), recombination layer studies (Yoon et al., 2020), and bottom Si cells structure (Wu et al., 2017). In 2020, Xu et al. achieved a high PCE of 27% in 2T monolithic tandem with an area of 1 cm^2^.

A few excellent reviews have been published to summarize the rapid development of PVSK tandem solar cells. Anaya et al. published a review discussed the development on ABX_3_ PVSK materials for tandem cells, acting as the active material either in top subcells or bottom subcells (Anaya et al., 2017). There is also another review emphasizing the important role of the Crystalline-Silicon (c-Si) bottom cell with different passivation methods for PVSK/Si tandem cells, which provides guidance for developing high performance tandem cells (Yan et al., 2019). In 2018, Leijtens et al. highlighted the great potential of PVSK tandem solar cells with low costs to reach solar-to-electricity conversion efficiencies far above those of single-junction solar cells, and also discussed future research directions to develop tandem solar cells which could exceed the proof-of-concept stage (Leijtens et al., 2018a). These reviews provided detailed discussions on top-cells or bottom-cells of tandem solar cells, but still there were few reports focusing on detailed progress for monolithic PVSK/Si tandem solar cells.

Herein, we present a review on the progress of PVSK/Si tandem solar cells in a pragmatic perspective that is related to material design, processing, coupling with c-Si subcells, and cost consideration. We start from the top transparent electrodes to discuss the research progress in most configurations that have been adopted. Firstly, we introduce the research progress of transparent electrodes materials focusing on their fabrication methods and their advantages and limitations in tandem devices. We then discuss the composition and processing of the PVSK film used in tandem devices. It is worth noting that the bandgap of PVSK subcell in tandem cells should be larger than that of single-junction devices. In addition, the recombination layer and the bottom subcell c-Si are introduced in detail. Afterward, we provide a thorough retrospect of cost analysis of PVSK/Si tandem devices. Last, we finish with an outlook sharing our view on future paths of PVSK-based tandem cells development for the PVSK research community to follow, and we accept that such strategies could push the efficiency of PVSK-based tandems toward new horizons.

## Top Electrodes

In a 2T PVSK/Si tandem solar cells, only one front transparent electrode is required according to the following requirements. Firstly, the process of materials deposition has negligible damage to the subcells. No reaction with the beneath layer is allowed while the energetic atom is producing during deposition. Otherwise, an extra electrode buffer layer is necessary to protect the carrier transport layer. However, this layer might induce current loss. Secondly, the electrodes must be high-transmittance in the infrared and visible spectrum region so that the bottom cells could produce photocurrent more effectively. In addition, parasitic absorption and reflection loss in this contact layer should be inhabited to increase the photocurrent. More importantly, apart from optical transmittance and electron collection, other parameters such as band-gap alignment, scale-production and costs should also be taken into consideration. Based on the above-mentioned requirements, the candidate materials could be divided into 5 categories.

Considering the low sheet resistance, the high transmittance in the near infrared and visible spectrum region, transparent metal oxides (TMOs) are thought of as promising electrode materials which could be deposited by sputtering. However, the transparent layer under the electrode might get damaged during the deposition process. This issue could be fixed by inducing MoO_x_ buffer layer. Precious metals such as Ag and Au are not a qualified choice due to their thickness, which makes it so that sunlight cannot penetrate to produce photocurrent. As a result, ultrathin metal electrode with a thickness (<10 nm) manufactured by evaporated is employed as the top electrode. Besides, silver nanowires are chosen as electrodes together with TMOs or transport layer to enhance charge carrier collection ability. Graphene with excellent optical, electrical (15,000 cm^2^ V^−1^ s^−1^ of carrier mobility) and mechanical properties is another promising material for electrodes, in particular for fabricating flexible devices grown by chemical vapor deposition (CVD). According to the high costs of precious metals as electrodes, gold, indium, and silver free electrode strategy is proposed, which coats a carrier transport layer [poly(3,4-ethylenedioxythiophene):poly(styrenesulfonate) (PEDOT:PSS), NiO] on the polyethylene terephthalate (PET).

### TMOs

TMOs are promising materials for top electrodes on account of their low sheet resistance, high conductivity, high transmittance in near infrared and visible spectrum region. In addition, TMOs including indium tin oxide (ITO), indium zinc oxide (IZO) and doping with transition metals which could improve the properties of top electrodes.

#### ITO

**Figure 1:**
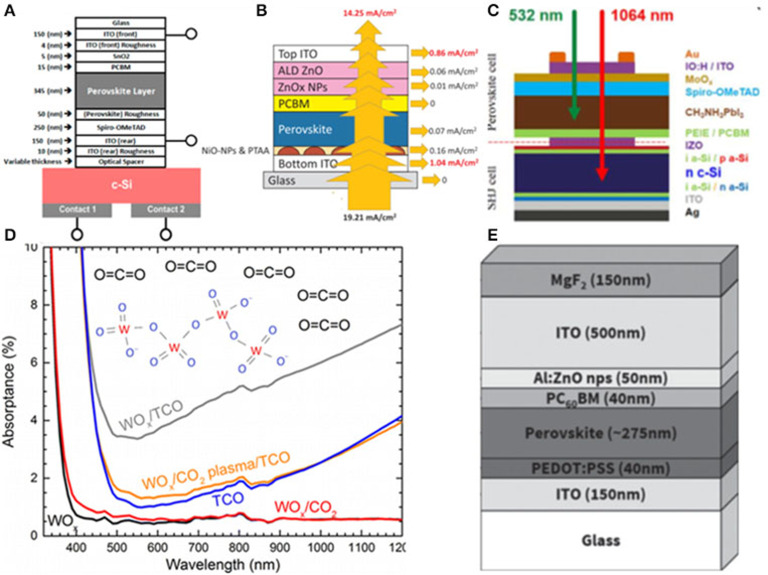
**(A)** Device structures of the 2T PVSK/Si tandem solar cell (reproduced from ref. 19, copyright 2017, Nature Energy). **(B)** Detailed optical loss analysis of ST-PSCs in NIR (800–1,200 nm). ITO layer thickness is set at 140 nm (reproduced from ref. 20, copyright 2018, Elsevier). **(C)** Schematic of the monolithic PVSK/Si tandem cells (reproduced from ref. 10, copyright 2016, ACS). **(D)** Absorption spectra and tauc plots of ~10 nm-thick evaporated MoO_x_, WO_x_, and V_2_O_x_ layers on glass (reproduced from ref. 21, copyright 2016, ACS). **(E)** Illustrative schematic of the device architecture showing the ITO electrode encapsulation layer (reproduced from ref. 22, copyright 2016, Wiley VCH).

Among all TMOs, ITO is one of the widely investigated materials for top electrodes. In 2017, Bush adopted 150 nm ITO as front contact to minimize the parasitic absorption as shown in Figure 1 (Bush et al., 2017). In order to reduce the reflection loss, Zhang et al. (2018) revealed the top electrodes of ITO implementing MgF_2_ on the top as antireflection coating, yielding an efficiency of 15.7% in single junction cell with a record near infrared transmittance of about 92% as well as the efficiency of 25.7% in the a 4-Terminal (4T) tandem cells.

Nevertheless, further optimization of efficiency is constrained by the deposition process which causes damage to the following transport layer. To address this, a buffer layer strategy was proposed by Song et al. (2016). They used MoO_x_ as buffer layer in monolithic PVSK/Si solar cells, and which had a bilayer front electrode of hydrogenated indium oxide (IO:H) and ITO. Eventually, they obtained an optimized efficient tandem device resulting from reducing carrier recombination.

Although the sputtering damage could be eliminated by employing MoO_x_ as a buffer layer, it may also induce re-coloration and parasitic absorption problems, which may seriously influence the efficiency. Werner et al. (2016a) proposed an approach that transition metal oxides worked as a buffer layer which was treated by oxidizing CO_2_ plasma to avoid any subsequent re-coloration during the cell fabrication process. In addition, the stacking of MoO_x_/WO_x_ could provide better electronic properties of MoO_x_, as well as reduce the parasitic absorption loss. As a result, transition metal oxides have been thought as another promising buffer layer material except the MoO_x_.

There were still some disadvantages for MoO_x_ worked as buffer layer such as the instability issue, which was mainly induced by the chemical reaction of MoO_x_ with the iodide in the PVSK. In general, MoO_x_ usually served as a buffer layer for the n-i-p structure. While, for the p-i-n structure, ZnO and SnO_2_ were commonly employed as buffer layer. In 2016, Bush et al. demonstrated an approach using solution-processed ZnO nanoparticles as the hole-blocking layer and sputtering buffer layer to replace MoO_x_ in inverted PVSK solar cells with sputtered 500 nm ITO directly onto the ZnO nanoparticles which achieved a low sheet resistance of ≈9.9 ohm sq^−1^ after annealing at 100°C (Bush et al., 2016). However, there was a large interfacial barrier existing between the ZnO and ITO layers preventing carrier extraction which might be eliminated via employing the aluminum doped (2 mol%) zinc oxide (AZO) nanoparticles worked buffer layer in tandem solar cells. Therefore, the efficiency was improved to 18.0% in the tandem solar cells, with a J_SC_ of 13.3 mA cm^−2^. In addition to ZnO, Eike Kohnen introduced the SnO_2_ buffer layer for the p-i-n structure. They fabricated 20 nm SnO_2_ by atomic layer deposition (ALD) technology. ALD is a kind of powerful-deposition technology for growing dense, conformal, and pinhole-free film, which would prevent damage being caused during the sputtering of the electrode. Based on the ALD SnO_2_ buffer layer, they yielded the efficiency of 26.0% as well as the J_SC_ of 19.5 mA cm^−2^. In short conclusion, the tandem solar cell that employs ITO as the top electrode has achieved high efficiency, and its fabrication technology has also been well-investigated and developed.

#### IZO

IZO with a high carrier mobility and low carrier concentration appears to be more suitable for transparent conductive electrodes compared to ITO. Moreover, the employing of ITO as the transparent electrode causes some problems such as severe parasitic absorption in a thicker ITO layer or high sheet resistance in a thinner ITO layer.

**Figure 2:**
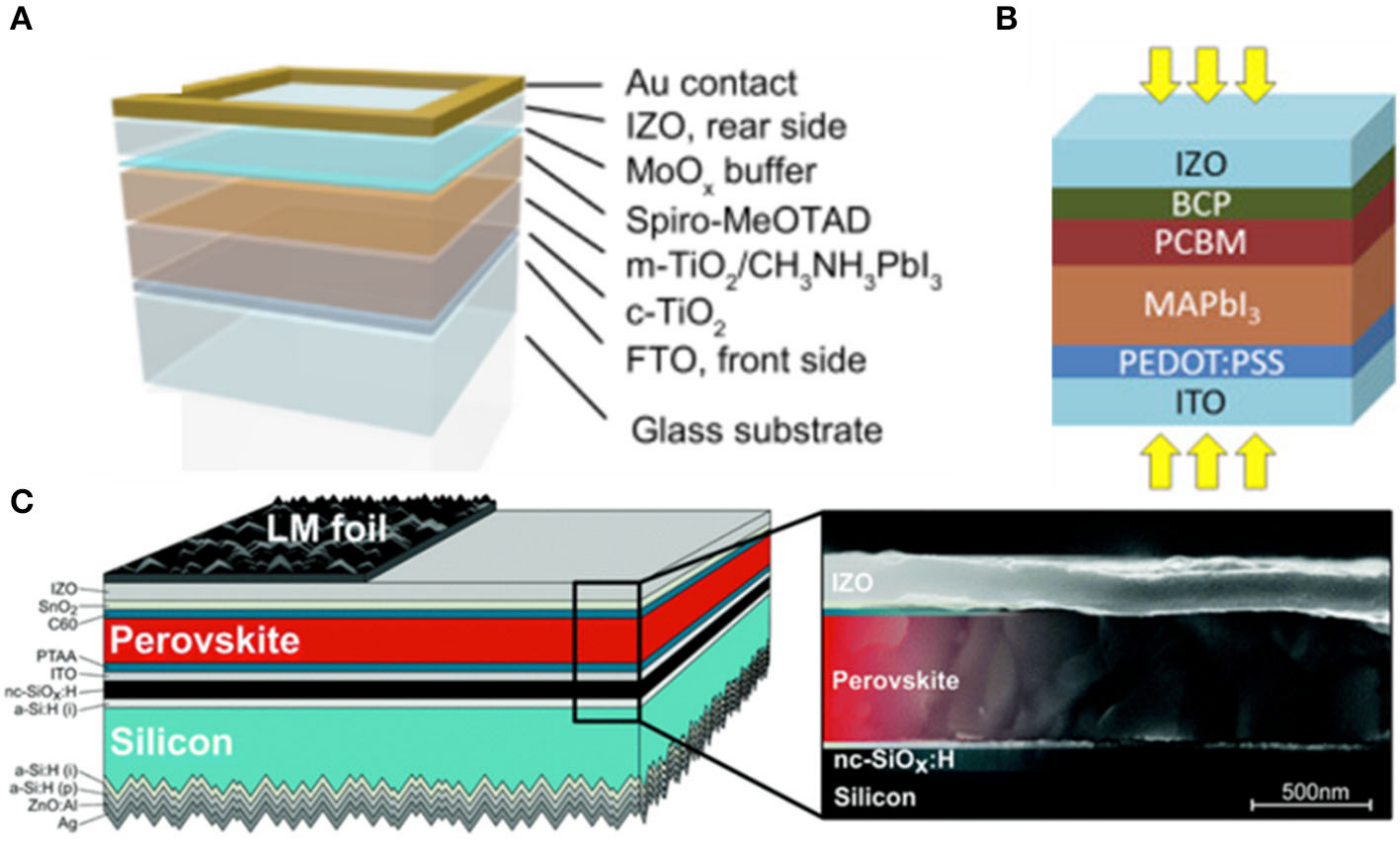
**(A)** Schematic illustration of a typical PVSK solar cell structure with a transparent rear electrode and Au contact (reproduced from ref. 23, copyright 2015, Elsevier). **(B)** Structure of semitransparent inverted PVSK solar cells (reproduced from ref. 24, copyright 2015, Elsevier). **(C)** Tandem solar cell device schematics of the experimentally realized architecture and SEM cross section image of the top cell with the layers as indicated (reproduced from ref. 25, copyright 2018, Royal Society of Chemistry).

In 2015, Werner et al. presented the rear electrode of sputtered IZO with a sheet resistance of 35 ohm sq^−1^, as shown in Figure 2 (Werner et al., 2015). They obtained an efficiency of 9% in the semitransparent PVSK solar cells without a buffer layer, and an efficiency of 10.3% with an MoO_x_ buffer layer. Such a strategy was also expected to be employed in the 4T tandem devices. Taking into account the inferiority of MoO_x_, Wahl et al. (2018) demonstrated an IZO layer sputtered at room temperature as the front electrodes in the invert PVSK solar cells, while the buffer layer was omitted by annealing at a moderate temperature to heal the damage in the following transport layer. They achieved an efficiency of 13% with negligible hysteresis, and a fill factor of 70%. Annealing may become another effective method to reduce the damage caused by sputtering without a buffer layer. In 2018, Jošt et al. presented detailed guidelines on how to reach high-efficiency tandem devices using a light trapping strategy (Jošt et al., 2018). They added textured light management foils on top of the IZO transparent contact to minimize the reflection in double-textured monolithic PVSK/Si tandem solar cells.

**Figure 3:**
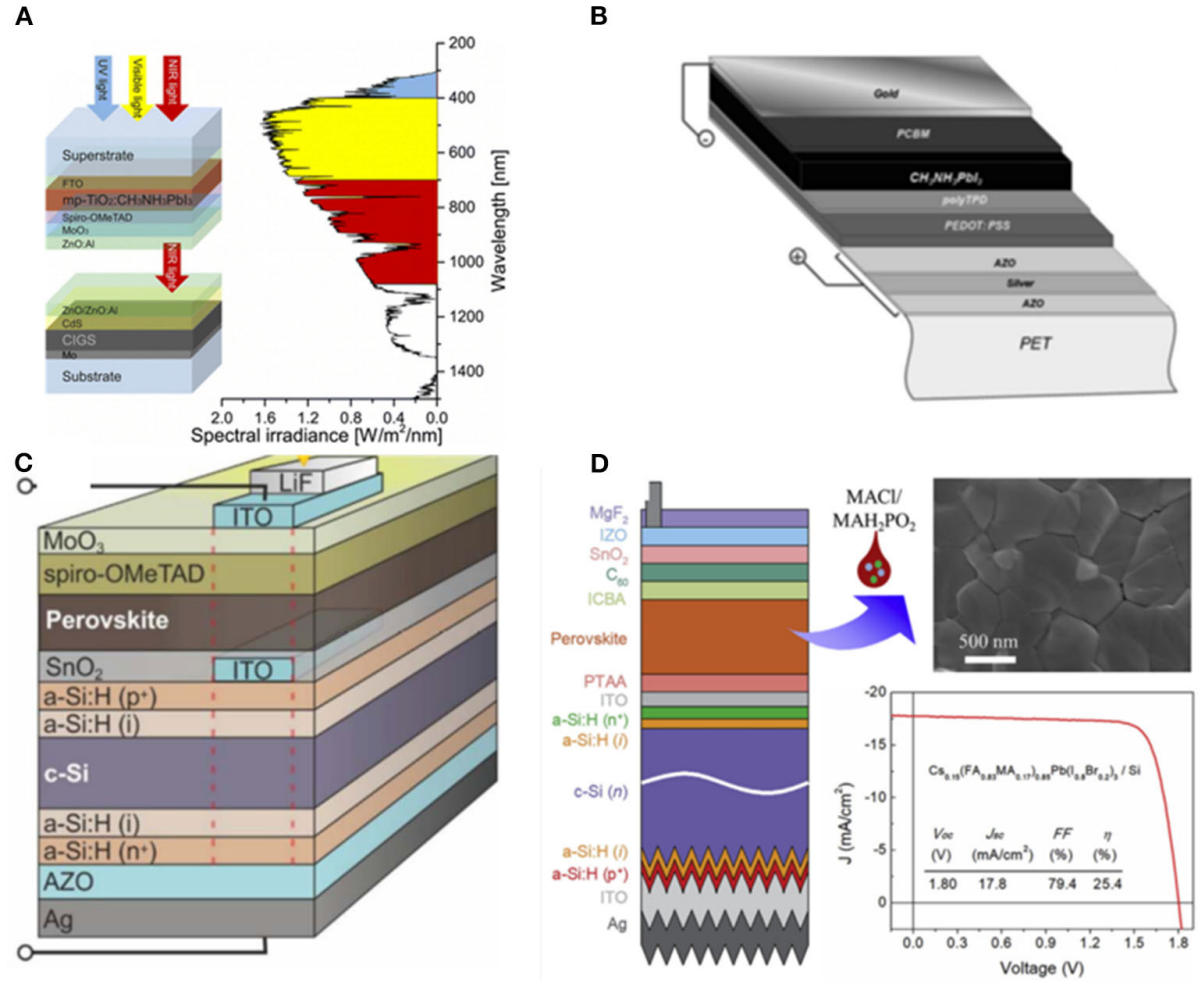
**(A)** Characterization of NIR-transparent PVSK cells (reproduce from ref. 26, copyright 2015, ACS). **(B)** Schematic layout of the flexible PVSK solar cell and chemical structure of the materials used as the electron and hole blocking layer (reproduced from ref. 27, copyright 2014, Royal Society of Chemistry). **(C)** Schematic device design of the Si heterojunction/PVSK tandem solar cell (reproduced from ref. 28, copyright 2016, Royal Society of Chemistry). **(D)** Schematic structure, J-V curves of Cs_0.15_(FA_0.83_MA_0.17_)_0.85_Pb(I_0.7_Br_0.3_)_3_/Si tandem device under forward and reverse scans with an inset photograph of the tandem device (reproduced from ref. 13, copyright 2017, Elsevier Inc.).

To reduce the carrier extraction barrier at the IZO interface, Kranz et al. (2015) configurated a sputtered ZnO:Al TMOs (IZO doped with aluminum) layer and a metallic grid on top of the MoO_x_ buffer layer with an average transmission of 71% for photons in the wavelength of 800–1,000 nm as shown in Figure 3. In this way, they were able to fabricate near-infrared-transparent PVSK solar cells with a PCE of up to 12.1%, as well as polycrystalline thin film tandem device with an efficiency of 19.5% combined with CIGS. The above results indicated that AZO could be taken as a better candidate for an electrode. However, AZO-incorporated devices also exhibited high resistance and low fill factor, which is not ideal, and could be improved by multilayer electrodes including a metal grid underneath the AZO layer as reported by Roldán-Carmona et al. (2014). They presented a three-layer structure of AZO, silver and AZO as electrode on PET substrate in flexible thin film solar cells, and obtained an efficiency of 7%. Apart from that, Albrecht et al. (2016b) fabricated the back contact of monolithic PVSK/Si tandem cells by sputtering 70 nm AZO and 200 nm silver, generating a stabilized power output of 18% and an open circuit voltage of 1.78 V. In addition, they presented an optical optimization strategy of Si cells to achieve a high efficiency device of 25% by replacing ITO with c-Si:H as a recombination layer. By calculation, they proposed a light trapping approach in electrodes that used a textured foil on top of the substrate of Si cells to reduce the reflection particularly in the long wavelength regime which could enhance the photocurrent.

Based on the multilayer stacking electrodes strategy, in 2018, Chen B. et al. (2019) demonstrated grain engineering via the introduction of two types of additives, MACl and MAH_2_PO, to improve the grain morphology and broaden the bandgap of PVSK with the electrode of Cu grid underneath IZO which could reduce the sheet resistance of electrodes as well as improve the fill factor, resulting in a high tandem V_OC_ of 1.80 V, high fill factor of 79.4% as well as PCE of 25.4%.

In conclusion, doping has obtained excellent PV properties with H, Zn and so on. However, In_2_O_3_:H could be damaged by water vapor diffusion. IZO may induce parasitic absorption because of its amorphous nature. Besides, other transition metals such as Mo, Ce, Ti, W and so forth that may also be dopant were not discussed in tandem cells due to their high annealing temperature, which was not compatible with the underlayer process. Aydin et al. (2019) sputtered zirconium oxide (ZrO_2_) doped indium oxide (IZRO) with 2 wt% ZrO_2_ as transparent electrode enabling high electron mobility of 77 cm^2^ V^−1^ s^−1^ and a low sheet resistance of 18 ohm sq^−1^ in 100 nm films. They also proposed that the crystallinity could be improved by Zr-doping resulting in a high conductivity and an improved efficiency of 26.2%. Moreover, Rucavado et al. demonstrated that the thickness of the film and annealing process may influence the microstructure of this layer and its carrier transport property. The IZRO annealing in neutral (N_2_) or reducing atmosphere (H_2_) remained mainly amorphous while annealing in air results in polycrystalline films with an average grain size ranging from 350 to 500 nm. Besides, they sputtered the IZRO with different thickness from 100 to 15 nm, with electron mobility of 100 and 50 cm^2^ V^−1^ s^−1^ for 100 and 15 nm films, respectively. On the other hand, films annealed in H_2_ ensured high carrier density but low carrier mobility. In a word, doping with H, Zn, Zr, translation metals (Mo, Ce, Ti, W) and so forth could improve the carrier mobility and the optical transparency, leading to a higher efficiency of the solar cell comparing with the pure ITO.

### Ag Nanowires

The TMOs may not be suitable for large-area and flexible photoelectric applications due to their high resistance, the low conductivity of the thick layer, as well as poor mechanical properties.

**Figure 4:**
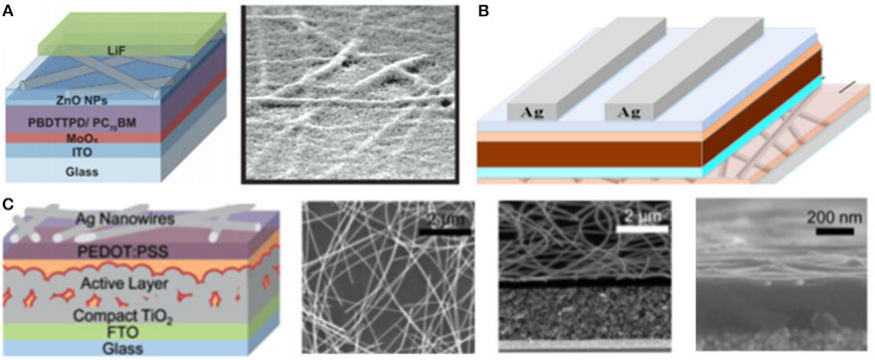
**(A)** Schematic of the semi-transparent cell architecture (reproduced from ref. 30, copyright 2013, Wiley VCH). **(B)** Schematic diagram of semitransparent ssDSC device (reproduced from ref. 31, copyright 2013, Wiley VCH). **(C)** Schematic illustration of the PVSK solar cell structure on an Ag NW/LGO composite electrode (reproduced from ref. 32, copyright 2019, MDPI).

To address this issue, Beiley et al. (2013) used networks Ag nanowires (Ag NW) as top electrodes in 2013 enabling transmission as high as 90% with sheet resistances below 13 ohm sq^−1^, which is far below the commercial ITO of 36 ohm sq^−1^, as shown in Figure 4 (De et al., 2009). Nevertheless, because of the corrosion of halogen species from PVSK, Ag NW based contact was not stable and their contact may not well-collect the charge carriers. Filling materials under Ag NW was explored. Beiley et al. proposed ZnO nanoparticles as filling materials for Ag NW mesh which reduced the sheet resistance from 16 to 14 ohm sq^−1^. They combined Ag nanowires with ZnO nanoparticles as top electrodes, achieving an efficiency of 5% in single junction cells. In addition, they anticipated that by employing such a strategy, the efficiency of GIGS/PVSK solar cells may reach as high as 20.7%. Moreover, Margulis et al. (2013) developed a Ag NW/PEDOT:PSS electrode via spraying method, achieving >92% transmittance in solid-state dye-sensitized solar cells (ssDSCs). The efficiency (3.6%) of the cell was just 0.1% less than the standard ssDSCs employing the evaporated silver electrode. This approach provided important guidance to realize hybrid tandem PV (HTPV) with high efficiency. Recently, Chen H. et al. (2019) used large size graphene oxide sheets with 60 μm thickness to form a protective barrier to cover the cracks of Ag NW in 2019. The composite electrode of Ag NW/Graphene Oxide maintained 98.4 and 95.1% average transmittance of the original contact and yielded a power conversion efficiency of 9.62%. Generally speaking, Ag NW is a promising candidate for flexible and large-area PV devices in the future.

### Ultrathin Metal Electrode

**Figure 5:**
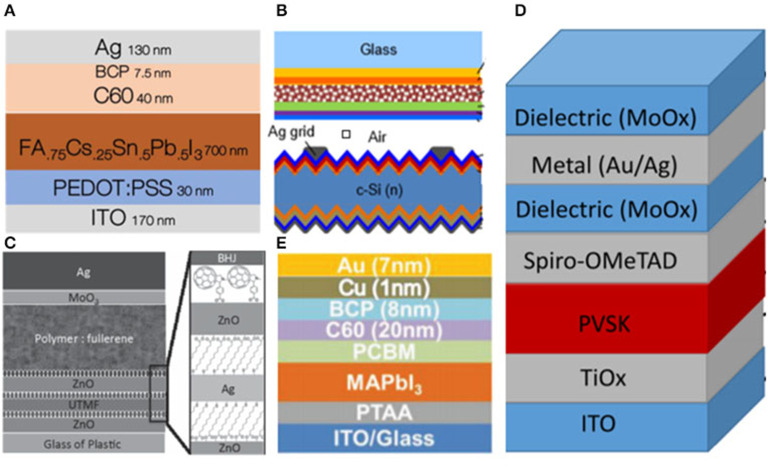
**(A)** Device schematic of the single junction solar cell (reproduced from ref. 33, copyright 2018, Royal Society of Chemistry). **(B)** Schematic of the mechanically stacked 4T tandem (reproduced from ref. 34, copyright 2014, Royal Society of Chemistry). **(C)** Schematic drawing of the PSC devices and the molecular structure of MUA and C_60_-SAM employed for interfacial modifications (reproduced from ref. 35, copyright 2014, Wiley VCH). **(D)** Device structure and performance of top illuminated PVSK solar cell (reproduced from ref. 36, copyright 2015, ACS). **(E)** Schematic drawing of tandem cells (reproduced from ref. 37, copyright 2016, Wiley VCH).

The manufacturing process of Ag NW is complex, and variability in the applied force may cause shorting as well as incomplete electron transfer, resulting in limited efficiency. Ultrathin metal usually has a thickness of <10 nm, combining the merits of high conductivity, well-mechanical-flexibility and ease of manufacturing without a pattern grid. In 2018, Leijtens et al. (2018b) fabricated 2T tandem cells with 130 nm Ag electrodes attaining external quantum efficiencies >80% in the near infrared region in Figure 5.

Nevertheless, this efficiency was far below single subcell, even though the tandem solar cell was composed of high-efficiency subcells with record V_OC_ and FF values. This may be attributed to the severe current losses because of reflection and parasitic absorption. Besides, precious metals such as Ag could not grow continually for its poor wettability, which could be solved by using TMOs as seed layer to grow metal oxide/thin metal/metal oxide tri-layer structure (MO/M/MO). Zou et al. (2014) reported a tri-layer structure in Ag films using ZnO as a seed layer and a fullerene-based self-assembled monolayer to achieve ohmic contact and improve charge collection. Thereby, the solar cells exhibited low surface roughness, high transparency and low sheet resistance (8.61 ohm sq^−1^). Based on the model of dielectric/metal/dielectric (DMD) (MoO_x_/Ag/MoO_x_), Yang et al. (2015) developed an ultrathin gold seed underneath the silver layer as the front electrodes with high conductivity and transparency. They delivered the efficiency of 15.5% with neglected V_OC_-loss and FF-loss in 4T PVSK/CIGS tandem cells. Chen et al. (2016) demonstrated an ultrathin Cu (1 nm)/Au (7 nm) metal electrode in semitransparent PVSK solar cells, attaining the PCE of 16.5%. Besides, they connected this cell with Si achieving the efficiency of 23.0%. Moreover, WO_3_/Ag/WO_3_ was also used as the top electrode in planar devices (Loper et al., 2015). In addition to WO_3_/Ag/WO_3_, Liang et al. (2018) sputtered WO_3_/Ag/SnO_2_ (WAS) with aqueous soluble SnO_2_ nanoparticles to tune the band gap mismatch between electrodes and PVSK in 2018 achieving the efficiency of 14%. OMO top electrodes have shown great potential in the PV filed, and could be explored further.

### Graphene

In recent years, some non-metal electrodes such as graphene have been developed. It has a higher near-infrared transparency compared with the metal-based electrodes. Besides, the contact layer in tandem solar cells must possess high electrical conductivity and optimal transparency.

#### Pristine Graphene

**Figure 6:**
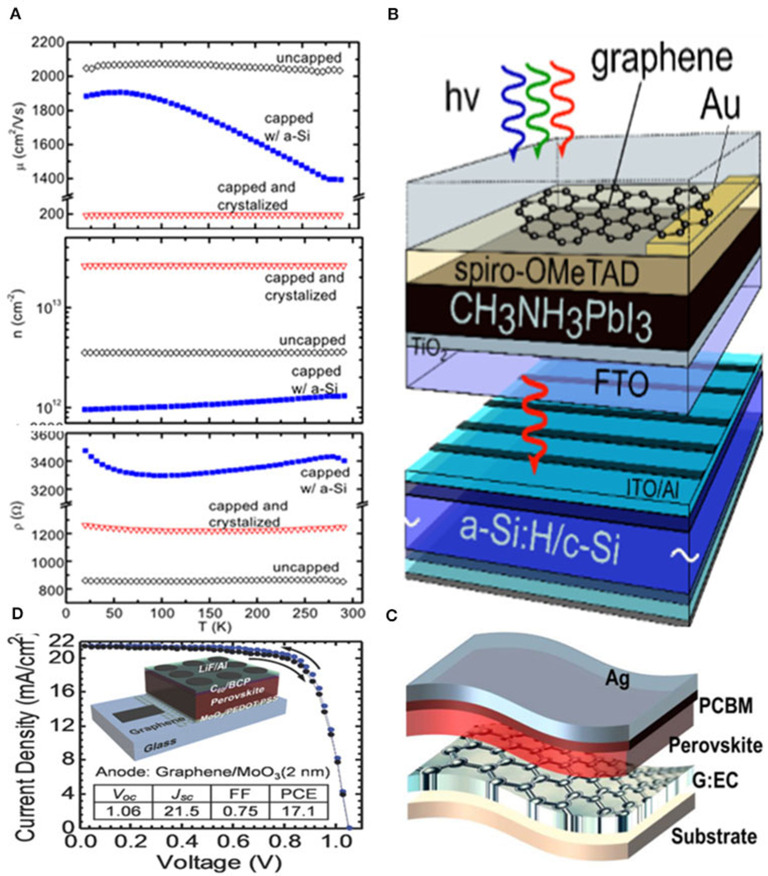
**(A)** Temperature dependence of Hall mobility, charge carrier density, and sheet resistance of bare graphene on a glass, capped with amorphous, and post-crystallized Si (reproduced from ref. 40, copyright 2013, AIP). **(B)** Simplified sketch of a four terminal tandem solar cell consisting of graphene based PVSK top solar cell and an amorphous c-Si bottom solar cell (reproduced from ref. 41, copyright 2015, ACS). **(C)** The device structure of PSCs based on G:EC electrode (reproduced from ref. 42, copyright 2019, Springer). **(D)** Schematic structure of the inverted MAPbI_3_ PVSK solar cells utilizing graphene as a transparent anode (reproduced from ref. 43, copyright 2013, Royal Society of Chemistry).

In 2013, Gluba et al. (2013) synthesized graphene films via the CVD method, resulting in charge-carrier mobilities of 2,030 cm^2^ V^−1^ s^−1^ at hole concentrations of 3.61 cm^2^ and transferred this film to Si-based devices, as shown in Figure 6. Besides, they found that the hole concentrations and charge-carrier mobilities would be influenced by the deposition and subsequent crystallization of Si as revealed by Raman backscattering spectroscopy and Hall-effect measurements. Soon, a graphene layer was applied into tandem cells. Lang et al. (2015) grew large-area single layer graphene by CVD and transferred this onto the PVSK solar cells attaining excellent optical transmission of 97.4% with a sheet resistance of 100 ohm sq^−1^. They have attained a PCE of 13.2% in 4T PVSK/Si tandem solar cells, and remarkable open circuit voltages of around 1 V in single cells, whose performance has reached the standard of Au electrodes already. In order to explore a manufacturing process which is more suitable for industrialization, an innovative and economic technology was developed by Yao et al. (2019), showing a strategy of solution-processed graphene which could satisfy the demand of both conductivity and transmittance for electrodes with the assistance of ethyl cellulose in the flexible PVSK solar cells resulting in a maximum efficiency of 15.71%. Yoon et al. (2017) reported CVD graphene as transparent conductive electrode on a thin polyethylene naphthalate (PEN) rather than glass, and induced MoO_3_ as hole-doping in 2016 to construct flexible PVSK-based solar cells. As a result, they achieved a PCE of 16.8% with no hysteresis and superior bending stability. Apart from graphene, Li et al. (2014) proposed the CH_3_NH_3_PbI_3_/CNTs PVSK solar cell with direct bypassing energy consuming vacuum process of carbon nanotube network without hole transport layer as well as Au electrode attaining the efficiency of 6.29%. They also incorporated spiro-OMeTAD as a hole transport layer and improved the efficiency up to 9.90%. It should also be noted that this property could be further improved by purification or chemical doping to increase the conductivity and work function.

#### Doped Graphene

In order to improve the photoelectrical properties of electrodes such as conductivity and absorption, a doping strategy was adopted in the carbon-based materials. The doping graphene has a sheet resistance of 10 ohm cm^−2^, while it is 377 ohm cm^−2^ for the pure graphene (De and Coleman, 2010). Usually, the dispersion of carbon nanotubes could be obtained by covalently modifying or non-covalently modifying and including a polymer. Raïssi et al. (2015) demonstrated single wall carbon nanotubes embedded in a copper-phthalocyanine derivative (TSCuPc) on a PET substrate as top electrodes in 2015 achieving an efficiency of 3.2%. Moreover, this layer could also be employed as an electron transport layer achieving an efficiency of 7.4%. Besides, Chawla et al. (2017) designed graphene incorporated with ZnO nanocomposite in the a-Si:H/lc-Si:H tandem cells achieving better photon management. They increased the J_SC_ by 41.8% through varying the thickness of graphene/ZnO. In addition to high efficiency, long term stability was also important for tandem cells which could be obtained by doping the electrode. Bhandari et al. (2020) proposed carbon electrode doping with varied concentrations of WO_3_ nanoparticles possessing high stability in the air. In conclusion, introducing graphene and their ramification as top electrodes could reduce manufacturing costs which is beneficial for the industrialization of tandem cells.

### Precious Metals Free Conductors

The indium and precious metals free transparent conductor have been developed recently, owing to the reaction of precious metals (such as silver, gold, and indium) with any residual free halide from the PVSK. They demonstrated the stabilized power conversion efficiencies of 7.6 ± 1.0%. Bryant et al. (2014) proposed a laminate top electrode composed of corrosion proof Ni mesh electrode embedded in a PET film with a silver-free transparent conducting adhesive (TCA), attaining an efficiency of 15.5%. As a result, the employment of conducting polymer without any precious metal has shown great potential in achieving high efficiency cells.

### PVSK Absorber Layers

PVSK materials have been used as absorbing layers in solar cells due to their simple fabrication methods and superior electronic and optical properties (Jiang et al., 2015). One of the outstanding advantages in PVSKs with respect to other semiconductor materials is that they could tune their absorption onset by ion replacement, which results in tunable absorption edges within the wavelength range between 350 and 1,200 nm. Due to this, PVSK has been thought of as an ideal candidate for tandem cells. The wide-bandgap PVSK that was employed in the top subcell can be obtained by introducing Br^−^. However, this may lead to segregation and phase separation in the PVSK active layer with high Br^−^ content when under light illumination, which is detrimental for the PV performance and remains a challenge for the industrialization of high-efficiency tandem solar cells. In addition, it is imperative and important to develop convenient deposition methods to achieve high quality and conformal PVSK films on top of the textured Si.

### Bandgap Tuning

The PVSK can be expressed with a formula of ABX_3_, where A represents the organic amine cation which is usually MA^+^, B represents the metal cation such as the commonly used Pb^2+^, and X is the halide anion (Green et al., 2014). The bandgap of PVSKs can be tuned by composition engineering (Jesper Jacobsson et al., 2016). In general, the PVSK top-cell in the tandem structure utilizes the short wavelengths (higher energy) in the solar spectrum. Simulations also showed that a high-PCE PVSKs top-cell with bandgap of around 1.70–1.85 eV can boost the PCE up to 30% in a tandem structure (Lal et al., 2014; Yu et al., 2016). Therefore, it is important to develop high-PCE PVSK solar cells with suitable bandgap for tandem cell application. It should also be noted that some PVSKs with a relatively small bandgap of 1.55 eV also exhibit excellent PV performance in tandem devices.

#### X Site Substitution

In order to enlarge the bandgap of PVSKs, one of the common strategies is to partially substitute I^−^ by Br^−^ to form mixed halide PVSKs. In 2003, Tanaka et al. discovered the nature of excitons in MABr with a large bandgap, indicating that the bandgap of MAPbX_3_ could be tuned via substitution I^−^ with Br^−^ (Tanaka et al., 2003). In 2013, Noh et al. first showed that the bandgap of MAPb(I_1−x_Br_x_)_3_ PVSKs light absorbers increases with increasing Br^−^ content in precursors, resulting in a tunable bandgap from 1.55 to 2.3 eV (Noh et al., 2013), Similarly, several studies have also been reported to regulate not only the bandgap, but also photoelectric properties of PVSKs by introducing Br^−^, and it could be from MABr, FABr, and PbBr_2_ etc. (Jesper Jacobsson et al., 2016).

Although wide-bandgap PVSK can be achieved by tuning the halide composition, the segregation and phase separation were found in those mixed-halide wide-bandgap PVSK with high Br^−^ content under light illumination, which is detrimental to the PV performance. The light-induced halide segregation may lead to the formation of I-rich domains and Br^−^-rich domains in the MAPb(I_1−x_Br_x_)_3_ thin films, which could cause severe photo-instability problems in a mixed-halide wide-bandgap PVSK. Thus, there is an urgent demand to develop a facile route to stabilize wide-bandgap PVSK under illumination.

One strategy is to fabricate PVSK films with larger grain size to improve their photo-stability. Bi et al. first applied PTAA to facilitate the growth of PVSK films with large grains (Bi et al., 2015). By varying the grain size of the mixed-halide PVSK films using PTAA, they found that the MAPb(I_1−x_Br_x_)_3_ with a bandgap between 1.70 and 1.75 eV were stable under 1 sun continuous illumination for 30 min without notable degradation, as evidenced by the lack of splitting of XRD peaks (Hu et al., 2016). Furthermore, the mixed-halide PVSK with a bandgap of 1.70 eV also exhibited an enhanced PCE of 16.6%. In 2019, by adopting grain engineering, Chen et al. obtained PVSK films possessing large grain sizes of nearly 1 μm and a bandgap of 1.64 eV in top cell, achieving a 25.4% efficient PVSK/Si tandem device. For the encapsulated devices, they could retain 91.5% of their initial PCE after constant illumination of simulated AM 1.5 G light for 250 h (Chen B. et al., 2019). The enhancement of photo-stability by such a method can be explained by the suppression of ion-migration in a larger grain. Recently, Michael et al. reported efficient 1.67-eV wide bandgap perovskite top cells using triple-halide alloys (chlorine, bromine, iodine) to tailor the bandgap and stabilize the semiconductor under illumination. It suppressed the light-induced phase segregation in films even at 100-sun illumination intensity and <4% degradation in semitransparent top cells after 10,000 h of maximum power point operation at 60°C (Xu et al., 2020).

#### B Site Substitution

The Pb^2+^ cation, which occupies the B site in the PVSK structure, could be replaced by Cu^2+^, Sn^2+^, or Bi^3+^ (Ju et al., 2017; Liu et al., 2017; Williams et al., 2017). Among them, Sn^2+^ based PVSK has been investigated most intensively, but it absorbs in the near infrared region, and the bandgap can be decreased down to 1.17 eV. The smaller bandgap PVSKs were usually applied in the bottom cell of PVSK/PVSK tandem solar cells. As a result, B site substitution is not a common strategy for developing PVSK materials with large bandgap in the top cell of PVSK/Si tandem devices.

#### A Site Substitution

The MA^+^ cation may be replaced by formamidinium [CH(NH_2_${)}_{2}^{+}$, abbreviated as FA^+^]. Nevertheless, the larger size of FA^+^ cations may tilt the metal-halide octahedra, which will decrease the bandgap of the PVSK, i.e., the bandgap of MAPbI_3_ (1.6 eV) is smaller than that of FAPbI_3_ (1.45 eV) (Lee J. W. et al., 2015). Long-chain organic cations are also considered as promising candidates to substitute MA^+^ cation. For instance, Butamine [CH_3_(CH_2_)_3_(NH_3_)^+^, abbreviated as BA^+^] was a common employed cation to form 2D PVSK with large bandgap (Smith et al., 2014). The bandgap of two-dimensional (2D) PVSK [CH_3_(CH_2_)_3_(NH_3_)_2_(CH_3_NH_3_)_n−1_Pb_*n*_I_3n+1_] increases with the decreasing *n* value, from 1.53 eV of CH_3_NH_3_PbI_3_ to 2.24 of eV CH_3_(CH_2_)_3_NH_3_)_2_PbI_4_ (Stoumpos et al., 2016). The most commonly used 2D PVSK in PV application is CH_3_(CH_2_)_3_NH_3_)_2_(CH_3_NH_3_)_3_Pb_4_I_13_, which exhibits a bandgap of 1.65 eV and it is suitable for the top-cell of tandem solar cells (Tsai et al., 2016; Liu et al., 2020a,b). In addition, 2D PVSK films exhibits excellent humidity stability owing to their long organic chains [such as CH_3_(CH_2_)_3_NH_3_)^+^] compared totheir 3D counterparts. In early studies, 2D PSCs showed poor PCEs of only 4–5% due to its low carrier mobility and short diffusion length, which were caused by their large size spacers. In 2016, Tsai et al. introduced a pre-heating deposition method to grow 2D PVSK films with the orientation of inorganic framework perpendicular to the substrates, and thus facilitated the charge transport, resulting in an enhanced PCE of 12.5% with no hysteresis (Tsai et al., 2016). The unencapsulated 2D PVSK devices retained more than 60% of their efficiency after 2,250 h under constant and standard (AM 1.5G) illumination. Notably, for encapsulated devices, they almost showed no degradation under constant AM1.5G illumination or humidity (Tsai et al., 2016). However, the PCE of 2D PVSK devices were still falling behind their 3D counterparts.

Cs^+^ cations has also been employed in PVSK successfully. The bandgap of all inorganic PVSK CsPbI_1−x_Br_x_ changed from 1.73 eV (CsPbI_3_) to 2.3 eV (CsPbBr_3_) by varying the halide. The rapid development of inorganic PVSKs-based solar cells have given rise to an inspiring record PCE from the initial 6.7% to nowadays 17.75% (Liang et al., 2016). In 2018, McGehee's et al. found more Cs at A site rather than more Br at the X-site to raise bandgap is more ideal as it improved V_OC_. They obtained the perovskites with bandgap of 1.68 and 1.75 V and high efficiencies of 17.4 and 16.3%, respectively (Bush et al., 2018). However, the inorganic PVSK is unstable under ambient conditions, it would rapidly degrade from the cubic black phase to the undesirable orthorhombic yellow phase, which deteriorates the cells PV performance.

In conclusion, PVSK is a promising candidate for top subcell materials in tandem solar cells, as it could satisfy the bandgap requirement via simple composition engineering, while this is not available in many other optoelectronic materials.

### Film Processing

PVSK films employed as active layers in solar cells could be fabricated by spin coating, dip coating, gas-quenching, thermal co-evaporation or vapor phase conversion. Among all reported methods, spin coating was thought of as the dominated one, since it could regulate a reproducible and better film quality than other methods. These techniques make the integration of PVSK films on flat substrate in tandem devices easy. However, for textured substrate, the solution-processed spin coating method remains a challenge. Therefore, a hybrid two-step deposition method combining sequential co-evaporation and spin-coating was developed to form a conformal PVSK layer on the micrometer-sized pyramids of textured monocrystalline Si.

#### PVSK Layer Processed on Flat Substrates

Gas-quenching method: Almost simultaneously with the report of the solvent quenching method, the gas-quenching (GQ) method was developed. Instead of using anti-solvent, in the GQ method, a flow of nitrogen gas was employed to facilitate the evaporation of precursor solution during one-step coating. Smooth films with densely packed grains can be rapidly achieved after annealing. In 2016, an extensive study related to efficient multi-cation and multi-anion PVSK solar cells was reported. Such a method has been widely used in fabricating PVSK layer in tandem devices. In 2020, McGehee and his team reported efficient 1.67 eV wide-band gap PVSK top cells with triple-halide alloys (chlorine, bromine, iodine) adopting the GQ method. The device exhibited a two-time enhancement in terms of carrier lifetime and charge-carrier mobility comparing to controlled ones. This may be attributed to the enhanced solubility of chlorine by replacing some of iodine with bromine to shrink the lattice parameter. More importantly, light-induced phase segregation in PVSK films was suppressed significantly even under 100-sun illumination intensity. They achieved the PCE of 2T monolithic tandem cells reached 27% with an area of 1 cm^2^, and an enhanced stability with <4% degradation in semitransparent top cells after 1,000 h of maximum power point (MPP) operation at 60°C.

Solution spinning coating: The solution spin coating method has been widely used in fabricating PVSK films. By adopting this method, in 2016, Rech and his team first fabricated monolithic tandem cells with 18% efficiency (Albrecht et al., 2016a). In 2019, Chen et al. combine two additives, MACl and MAH_2_PO_2_ in PVSK precursor, which significantly improve the morphology of the wide bandgap (1.74–1.70 eV) PVSK films, resulting in a high tandem V_OC_ of 1.80 V and improved PCE of 25.4%. In 2020, Shin and his team used the solution spin coating method to develop stable PVSK solar cells with a band gap of ~1.7 eV and PCE of 20.7%. Those cells could retain nearly 80% of their initial PCE after 1,000 h under continuous illumination. In this work, anion engineering such as phenethylammonium (PEA)-based 2D additives was found to be critical for controlling the structural and electrical properties of 2D PVSK passivation layers based on a PbI_2_-framework. As a result, the high PCE of 26.7% in a monolithic 2T wide gap PVSK/Si tandem solar cell was achieved by combing spectral responses of the top and bottom cells.

#### PVSK Layer Processed on Textured c-Si

Hybrid two-step deposition method: At present, the most reported monolithic PVSK/Si tandem devices are based on a single-side texturing configuration. A textured back side for enhanced light trapping property compared to a double-side polished c-Si device, and c-Si wafers with their front surface flat-polished to be compatible with the existed solution based PVSK fabrication processes. However, light trapping property in such a configuration is not ideal. Therefore, building up efficient tandems using double-side textured c-Si approach becomes imperative. In order to get conformal PVSK layers and cells on textured c-Si, Ballif and his team developed a hybrid two-step deposition method combining sequential co-evaporation and spin-coating to yield conformal PVSK absorber layers on the micrometer-sized pyramids of textured monocrystalline Si. Tandem devices had a high photocurrent of 19.5 mA cm^−2^. It was found that the pyramidal texture of the Si bottom cell could directly reduce the primary reflection loss and enhance light trapping in the infrared region, leading to a certified steady-state efficiency of 25.2%.

Solution-processed PVSK on textured c-Si: Depositing PVSKs via solution-processed techniques on top of micrometer-sized Si pyramids also exhibited several shortcomings: uncovered Si pyramids, shunt paths, and inefficient charge collection in films with variable thickness, etc. Recently, Sargent (Hou et al., 2020) and his team developed a high-quality micrometer-thick PVSK to cover the pyramids. The charge collection abilities in these thick films were also enhanced due to the improved drift and diffusion of photo-generated carriers. In this work, they combined solution processed, micrometer-thick, wide-band gap PVSK solar cells with pyramidal-textured c-Si bottom cells. This approach achieved a three-fold enhancement of depletion width in the PVSK semiconductor at the valleys of Si pyramids, thereby improving the carrier collection. To further increase the carrier diffusion length, they introduced a conformal surface-passivation strategy for rough surfaces by anchoring a self-limiting passivator on the wide-band gap PVSK surface. This method was also proved to suppress the undesired phase segregation. As a result, the as-fabricated PVSK/Si tandem solar cells exhibited a certified PCE of 25.7%, as well as enhanced stability after a 400-h thermal stability test at 85°C and a 400-h stability measurement under maximum power point tracking at 40°C. In 2020, Huang's group reported the blade-coated perovskites on textured silicon with pyramids <1 μm in height. A nitrogen-assisted blading process deposits a conformal hole transport layer and perovskite layer that fully covers the textured silicon. They achieved a perovskite/silicon tandem device with an efficiency of 26% on textured silicon (Chen et al., 2020).

In summary, the deposition method for PVSK film has been developed and investigated constantly, and high-quality polycrystalline films can now be obtained on both flat and textured substrates with high reproducibility. In PVSK/Si tandem solar cells, there are no technical difficulties for plat c-Si. But for textured c-Si, it still appears to be difficult to get conformal films with uniform thickness via sequential co-evaporation and spin-coating methods, in particular for textured monocrystalline Si with large micrometer-sized pyramids. It will be of great significant to investigate and develop more convenient and efficient deposition methods for PVSK films on the textured substrate in the near future.

## Recombination Layers

In a monolithic PVSK/Si tandem cells, the two subcells are connected with a recombination layer or interconnecting layer which could recombine completely opposite electrons and holes from the electron transparent layer and the hole transparent layer of top and bottom subcells, respectively. An excellent interconnecting layer should achieve a neglected voltage-loss. As a result, there are some requirements demanded for the recombination layer: The work function of the recombination layer must effectively collect electrons and holes form different transport layers, achieving minimal electrical losses; The recombination layer could be manufactured at the low temperature without damage to the underneath layer; The transmittance of the recombination layer must be high and should not influence the absorption of bottom cells to induce current-loss.

On account of the demands mentioned above, TMOs are excellent candidates, which possess the properties of high transmittance, low sheet resistance, and high conductivity. ITO, IZO, and AZO are all popular materials for recombination layers. Because of the low shunt resistance of TMOs, voltage loss would be induced. As a result, Si homogeneous materials are good candidates for the recombination layer including amorphous Si, p type Si, n type Si, hydrogenated microcrystalline Si (μc-Si:H) or doped-Si materials depositing by the low temperature plasma-enhanced CVD process with higher shunt resistances. In addition, doped organic molecular combined with underneath or upper transparent layer such as m-PEDOT:PSS/PH_1,000_/ZnO and Spiro-OMeTAD/PEDOT:PSS/PEI/PCBM:PEI could replace the materials mentioned above with a low temperature manufacturing process and low costs. However, adding an extra recombination layer would induce an inevitable current loss for devices. A strategy called recombination layer free is proposed by combining the electron transparent layer and hole transparent layer from different cells which could also simplify the manufacturing technology.

### Si-Based

**Figure 7:**
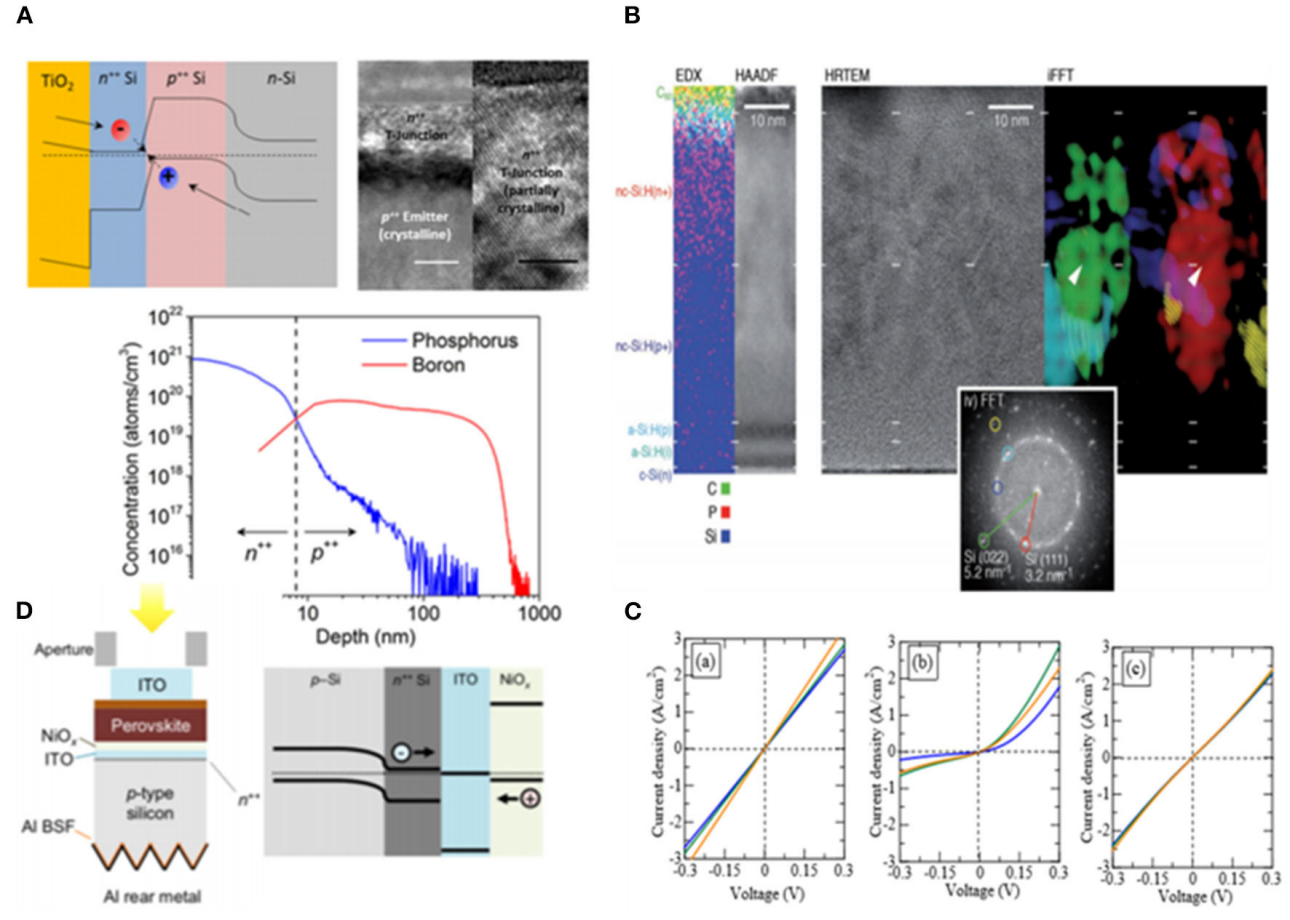
**(A)** Band diagram, TEM image, and SIMS profile of the PVSK/Si cell tunnel junction interface (reproduced from ref. 67, copyright 2015, AIP). **(B)** EDX map and corresponding STEM HAADF image of the recombination junction (reproduced from ref. 68, copyright 2017, Wiley ACH). **(C)** TRJ structure using p-Cu_2_O:N=n-a-Si:H, and TRJ structure using p-Cu_2_O:N=n-μc-Si:H (reproduced from ref. 69, copyright 2018, The Japan Society of Applied Physics). **(D)** Device structure of tandems with Al-BSF p-type Si bottom cells and structure of the ITO/NiOx recombination contact on a p-type Si cell (reproduced from ref. 70, copyright 2018, IEEE).

Si based materials with low lateral conductivity can reduce V_OC_-loss which could achieve high efficiency for tandem cells. The first monolithic tandem cells were fabricated by Mailoa et al. (2015), and Si-based tunnel junction was deposited via plasma-enhanced chemical vapor deposition (PECVD), resulting in the majority-carrier charges recombination effectively in tunnel junction, as shown in Figure 7. This is the first time that tandem cells were constructed, and the possibility for Si to work as recombination layer was proposed. In the end, they obtained the V_OC_ of 1.65 V and the device efficiency of 13.7%, while the performance of the device was restricted by high-temperature-deposition mesoporous TiO_2_ which should be eliminated. Also, low lateral conductivity of Si influenced further improvement of efficiency.

To solve the conductivity of Si, Sahli et al. (2018) constructed monolithic PVSK/Si heterojunction tandem cells with a recombination layer of nanocrystalline Si deposited by PECVD at a low temperature which could reduce parasitic absorption and reflection losses. Therefore, they achieved the efficiency of 22.7% while V_OC_ was 1,751 mV, J_SC_ was 16.8 mA cm^−2^ and FF was 77.1% for the area of 0.25 cm^2^. Moreover, doping could enhance the conductivity of Si proposed by Kim et al. (2018) via comparing two types of recombination layer of both n-type hydrogenated microcrystalline Si (μc-Si:H) and n-type Si with nitrogen-doped cuprous oxide (Cu_2_O:N) in resistance. They found that the first layer showed non-ohmic behavior while the second type of layer exhibited ohmic contact with a low resistance (3.9 × 10^−2^ ohm sq^−1^). This method proposed a brand-new direction to obtain high efficiency. However, the Si recombination layer could be influenced by the oxidation of SiO_x_ from the Si bottom cell during the fabrication of top cell. Hoye et al. (2018) sputtered 30 nm ITO incorporated with nickel oxide as recombination layer to protect from oxidation. As a result, TMOs have become prospective materials for recombination layers.

### TMOs Based

The manufacturing processing of TMOs as recombination layers is more convenient considering TMOs are a part of Si heterojunction cells (SHJ). Albrecht et al. (2016b) demonstrated that ITO worked as recombination layer in 2016 in monolithic PVSK/Si tandem cells processed at a low temperature attaining the efficiency of 19.9% with a short circuit current of 14 mA cm^−2^, a voltage of 1.78 V, and a FF = 79.5%. While the high absorption of ITO in the near-infrared spectrum reduced the efficiency of tandem cells which could be resolved by reducing the recombination thickness to enhance photocurrent. Albrecht et al. (2016a) reduced the thickness of the ITO recombination layer from 80 to 40 nm attaining a slightly enhanced absorption for bottom cells and achieving 17% efficient monolithic tandem cells. This slight improvement was achieved by eliminating the mismatch of current from the top and bottom solar cells. Furthermore, they optimized the thickness of n-type and p-type transport layers resulting in an efficiency of 28.4% and FF of 81% by assuming. Also, Albrecht et al. (2016a) optimized the recombination layer thickness of ITO yielding a stabilized power output of 17%.

**Figure 8:**
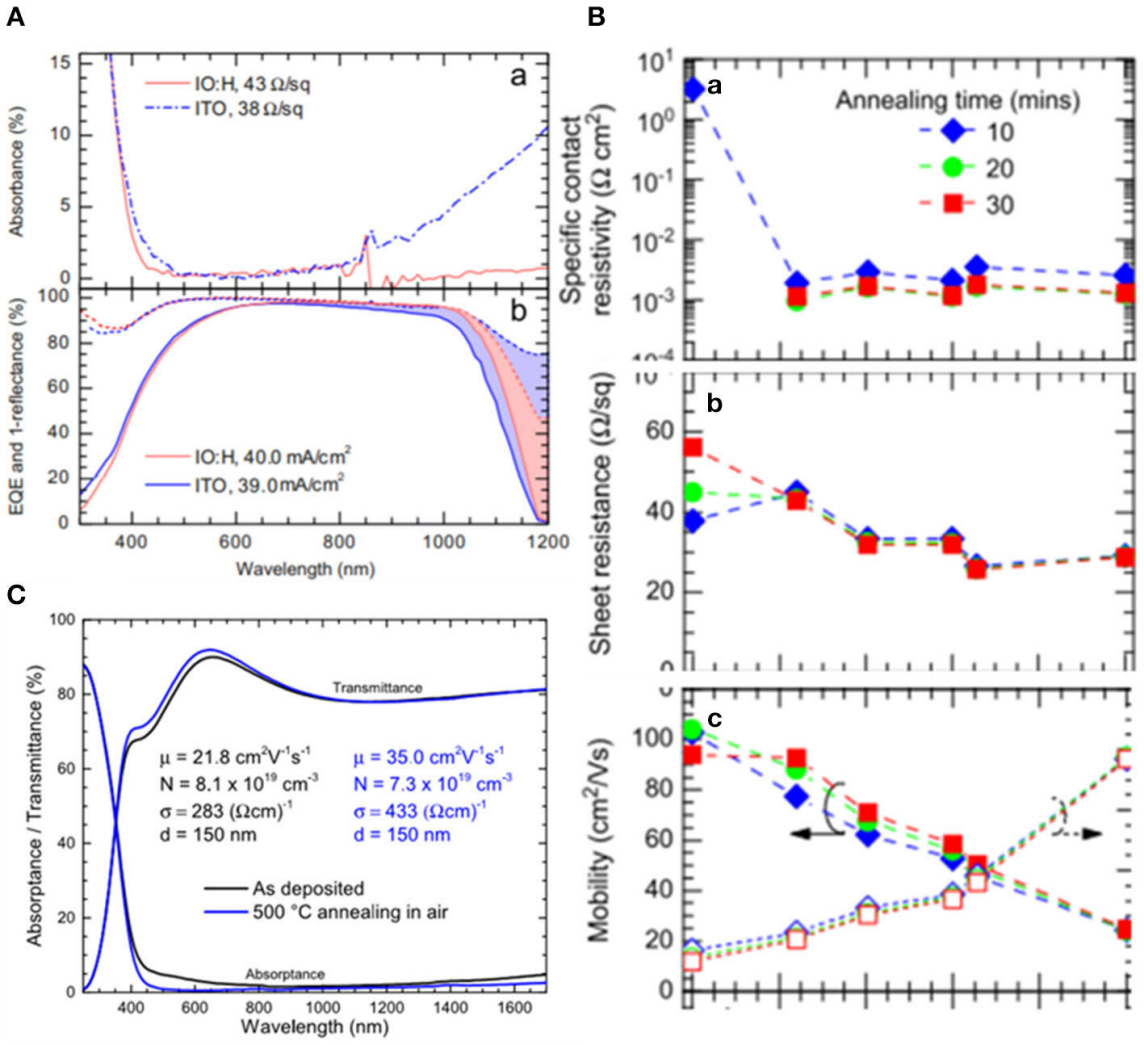
**(A)** Absorbance spectra, external quantum efficiency (solid), and 1-reflectance (dashed) spectra of IO:H and ITO films on glass (reproduced from ref. 10, copyright 2013, Elsevier). **(B)** Specific contact resistivity, sheet resistance, and mobility and free electron density of IO:H/ITO bilayers on glass (reproduced from ref. 10, copyright 2013, Elsevier). **(C)** UV-vis-NIR spectrophotometric of a 150 nm thick ZTO layer on glass before and after annealing at 50°C in air. Hall Effect characteristics are also given. l¼ mobility; N¼ carrier density; r¼ conductivity; and d¼ thickness (reproduced from ref. 73, copyright 2016, AIP).

TMOs such as ITO suffered from low carrier mobility resulting in insufficient carrier recombination and voltage loss which could be solved by doping, as shown in Figure 8. Barraud et al. (2013) demonstrated that IO:H had an electron mobility over 100 cm^2^ V^−1^ s^−1^. Apart from IO:H, the property of high carrier mobility and low carrier concentration assisted IZO to become more competitive for the recombination layer. In 2018, Song et al. (2016) constructed a recombination layer of IZO in 2T PVSK/Si tandem cells using light beam induced current system to identify defects in cells during the process of photocurrent collection and generation. Werner et al. (2016b) presented PVSK/Si heterojunction monolithic tandem solar cells with the recombination of sputtered IZO layer deposited at room temperature reaching the efficiency of 19.2% with an aperture area of 1.22 cm^2^ and the efficiency of 21.2% with small area of 0.17 cm^2^. Also, they investigated the influence of thickness via varying the thickness of IZO from 25 to 70 nm and observing that the optimum situation was achieved by a 40 to 50 nm-thick IZO layer demonstrating the importance of light management. Taking into account the high fabricating process of 500°C, mesoscopic PVSK cells were restricted to be implemented into monolithic tandem cells. Werner et al. constructed IZO as recombination layer which could stand this temperature obtaining the efficiency of 16%. Also, by varying the thickness of this layer from 20 to 160 nm, they found that the larger contracts of refractive index between the transport layer and recombination layer could induce the internal reflection at this interface. It should be noted that TMOs have attained excellent PV properties because of their wide exploration.

### Organic Molecule Based

Some special organic molecular with high transmittance could replace TMOs as the recombination layer due to the TMOs working as a recombination layer with disadvantages, including that they have an optical loss in the near-infrared region of 800–1,000 nm as well as lateral current loss for their reduced shunt resistance.

**Figure 9:**
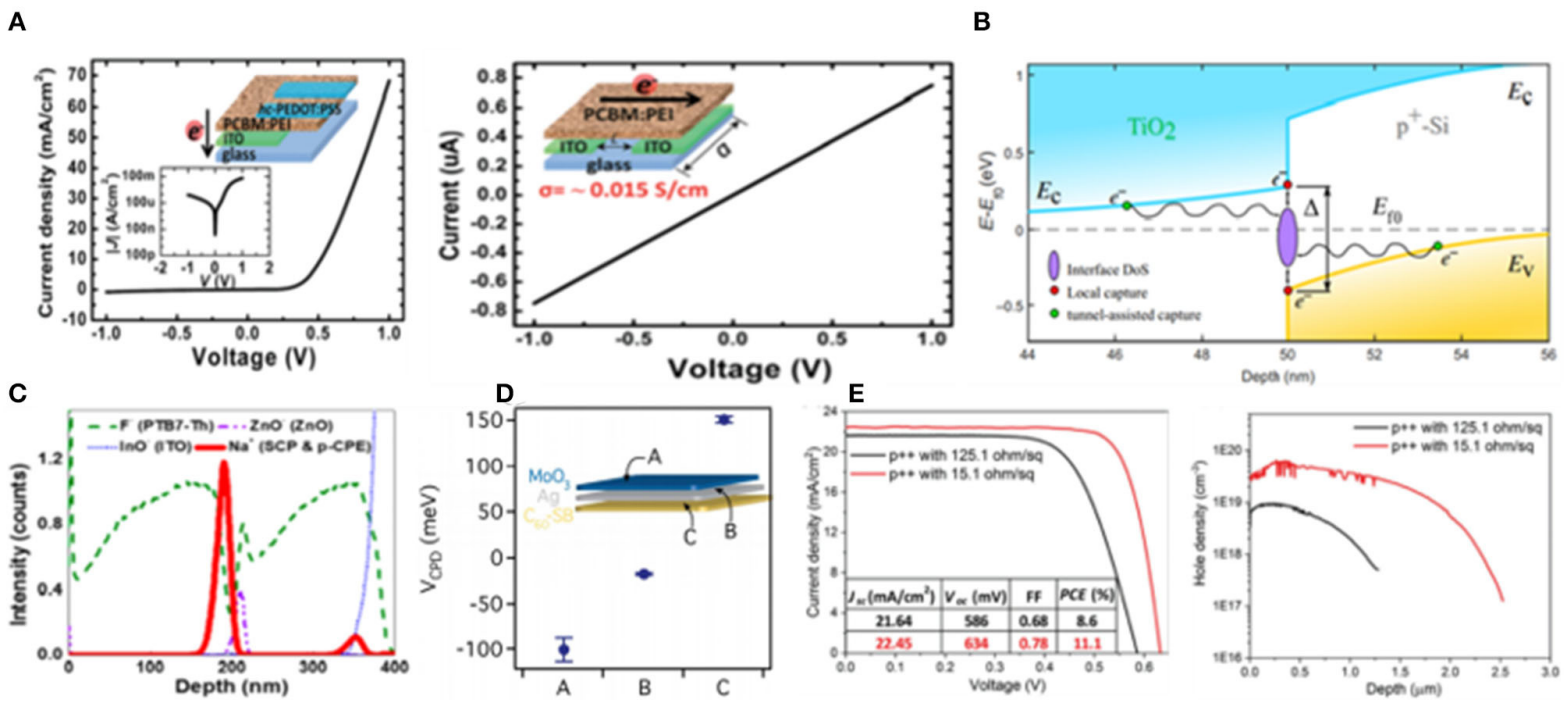
**(A)** J–V characteristics of the devices including the current flows vertically across the film and the current flows in parallel with the substrate (reproduced from ref. 74, copyright 2016, The Royal Society of Chemistry). **(B)** Contact behavior and simulated band diagram of TiO^2^/p^+^ -Si interfaces (reproduced from ref. 76, copyright 2018, Science Advances). **(C)** TOF-SIMS depth profile of the ITO/p-CPE:BHJ/ZnO/SCP/p^−^CPE:BHJ structure (reproduced from ref. 58, copyright 2016, ACS). **(D)** Surface potential (VCPD) of the recombination layers [C60-SB (30 nm)/Ultrathin Ag (10 nm)/MoO_3_ (10 nm)] (reproduced from ref. 75, copyright 2016, ACS). **(E)** J–V curves and electrochemical capacitance–voltage measurements of Si bottom cells that have different p^++^ emitter profiles (black for 125.1 ohm sq^−1^ and red for 15.1 ohm sq^−1^ (reproduced from ref. 78, copyright 2018, The Royal Society of Chemistry).

In 2016, Jiang et al. (2016) developed the recombination layer composed of spiro-OMeTAD/PEDOT:PSS/PEI/PCBM:PEI which could be manufactured with an all-solution method as shown in Figure 9. Eventually they obtained 2T tandem solar cells with a V_OC_ of 1.89 V, which was close to the sum of two subcells with no voltage loss. This work inspired the application of organic molecules as a recombination layer in tandem solar cells. Unfortunately, some organic molecules were restricted because of their unstable properties, for example, PEDOT:PSS was not suitable as a p-type recombination layer because it was unstable due to its acidic nature. In order to avoid this issue, Lee et al. demonstrated the stable and high efficiency tandem devices employing self-doped conducting polymer (SCP) combined with ZnO nanoparticles worked as a recombination layer without PEDOT:PSS which achieved the PCE of 10.2% as well as extended long-term stability (Lee et al., 2016). Further, they provided a strategy for the recombination layer using SCPs to achieve efficient and stable tandem solar cells by discussing the impacts of pCPE:BHJ nanocomposite on enhancing the tandem performance. Besides, a multilayer stacking recombination layer strategy was constructed by Liu et al. (2017) using a graded recombination layer containing a zwitterionic fullerene, silver (Ag), and MoO_3_ attaining the efficiency of 16.0% in tandem PVSK/polymer solar cells.

A method called no-extra recombination layer was proposed by Zheng et al. (2018) in order to avoid the optical and electrical loss in recombination layer. They demonstrated the monolithic PVSK/homojunction Si tandem solar cells for the first time with no-extra recombination layer while dual-function SnO_2_ served as an electron transport layer as well as an interface layer whose poor lateral conductivity may be repaired by the p^++^ emitter in the Si cell. In the end, they yielded a PCE of 21.0% with an area of 4 cm^2^ under reverse-scanning with a V_OC_ of 1.68 V, a J_SC_ of 16.1 mA cm^2^ and a high FF of 78%. They also achieved a steady-state PCE of 17.1% on a large area of 16 cm^2^. Moreover, Shen et al. (2018) proposed the strategy using n-type TiO_2_ and Si manufactured by atomic layer deposition worked as recombination layer attaining the efficiency of 24.1% for passivating contact heterojunction PVSK/Si tandem cells. In summary, this strategy displayed high performance without introducing extra interlayer which was cost-effective.

## c-Si Subcells

**Figure 10:**
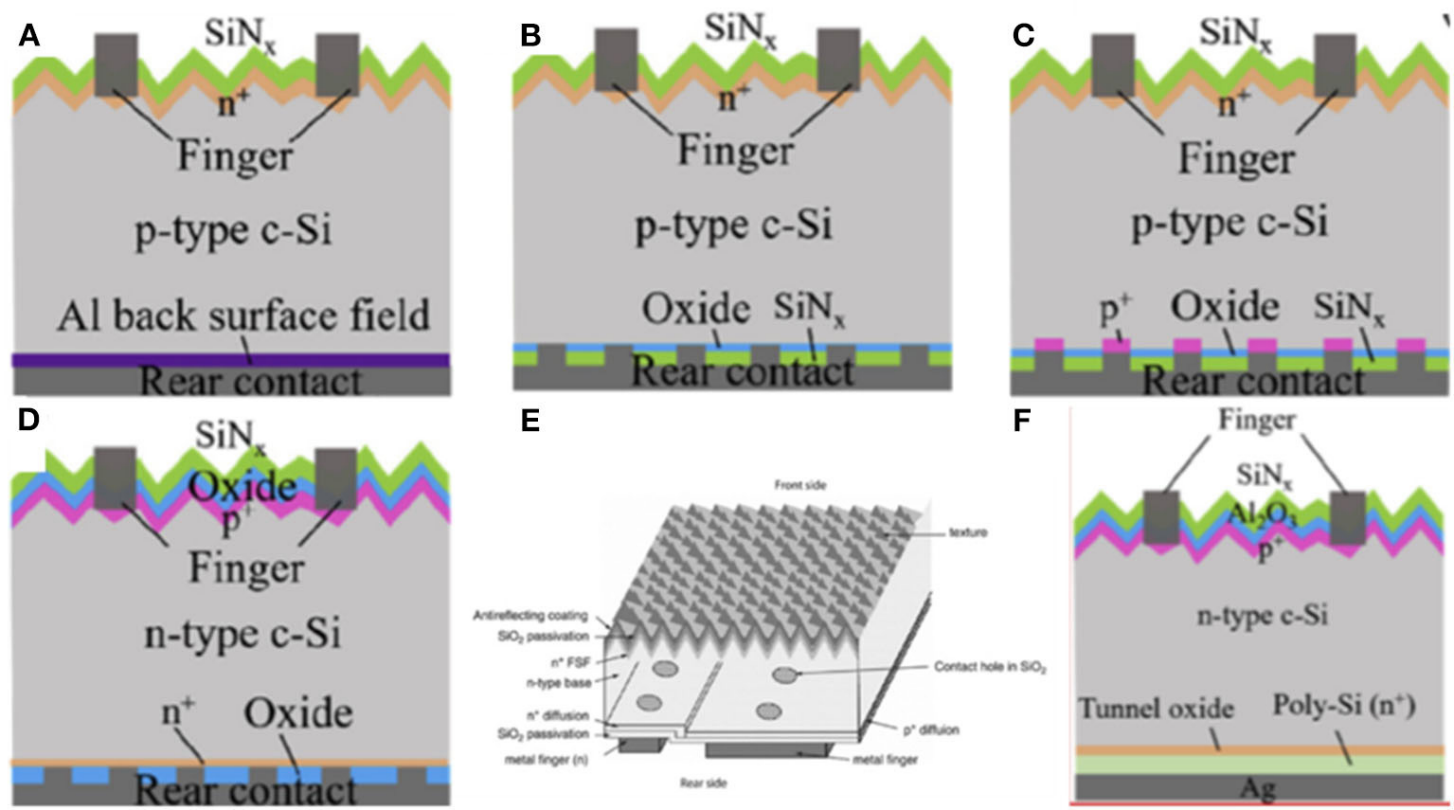
Schematic view of **(A)** the standard full-area Al-BSF cell, **(B)** the PERC device, **(C)** the PERL cell, and **(D)** the PERT cell (reproduced from ref. 77, copyright 2019, Elsevier). **(E)** IBC (reproduced from ref. 17, copyright 2015, Wiley VCH). **(F)** TOPCon.

The c-Si solar cell is the pre-dominant PV technology with the market share more than 90% thanks to its appropriate bandgap of 1.12 eV, high stability and non-toxicity. The standard all-area aluminum back surface filed (Al BSF) Si solar cells are constructed including (1) p-type wafer doped with boron and diffused by phosphorus as the front emitter reaching an efficiency of 18.5% in Figure 10A (Lee Y. et al., 2015). Besides, (2) a front metal grid and antireflection coating are printed on the emitter and (3) Al BSF is pasted at the back of the cell with back metal contact. In order to further improve the efficiency of Si cells, optical and electrical strategies have been adopted. Firstly, surface and interface passivation are developed to reduce recombination and minimize carrier loss. Secondly, advanced antireflection films and random pyramid structure are implemented to reduce optical loss. In addition, improving the quality of the Si wafer to enhance carrier diffusion length attained the minimized electrical loss. Therefore, highly efficient Si solar cells over 26% approaching to S-Q limit for their reduced electrical, optical and carrier loss have been achieved. Based on the passivation, they could be divided into homojunction Si with a high temperature tolerance (>400°C) and heterojunction Si often processed at low temperatures (<250°C). In order to further improve the PV properties of devices, tandem solar cells with an Si bottom subcell and PVSK top subcell have been constructed which could obtain high efficiency surpassing S-Q limit of single junction solar cells.

### Homojunction Si

Homojunction Si whose emitter was formed by diffusion was an excellent candidate for tandem bottom subcell. On the one hand, they have taken a great part in global Si market share of around 93%. On the other hand, process compatibility of high temperature tolerance of Si bottom subcell for 2T tandem cells should be considered for high-temperature-processed PVSK top subcell which made homojunction Si more suitable for tandem cells. They contained passivated emitter and rear cells with different diffused processing (PERX group) [passivated emitter and rear cells (PERC) in Figure 10B, passivated emitter and rear locally diffused (PERL) cells in Figure 10C, passivated emitter and rear totally diffused (PERT) cells in Figure 10D], interdigitated back-contact (IBC) in Figure 10E, tunnel oxide passivated cells (TOPCon) in Figure 10F and so on (Yan et al., 2019). The first monolithic PVSK/Si tandem cells was demonstrated by Mailoa with a homojunction Si subcell. They obtained an efficiency of 13.7% employing heavily doped n-type Si as tunnel junction.

#### PERX Family Cells

PERC Si was passivated with Si nitride (SiN_x_) on the rear oxide layer based on standard Al BSF Si. Then, this layer was locally removed to form local metal contact. In addition, PERL Si was improved based on PERC structure with heavily local boron diffusion on the rear surface. Furthermore, PERT Si possessed heavily local phosphorus doping in the n-type wafer with a local rear oxide layer by etching erosion. In conclusion, these three types of Si belong to PERX group.

**Figure 11:**
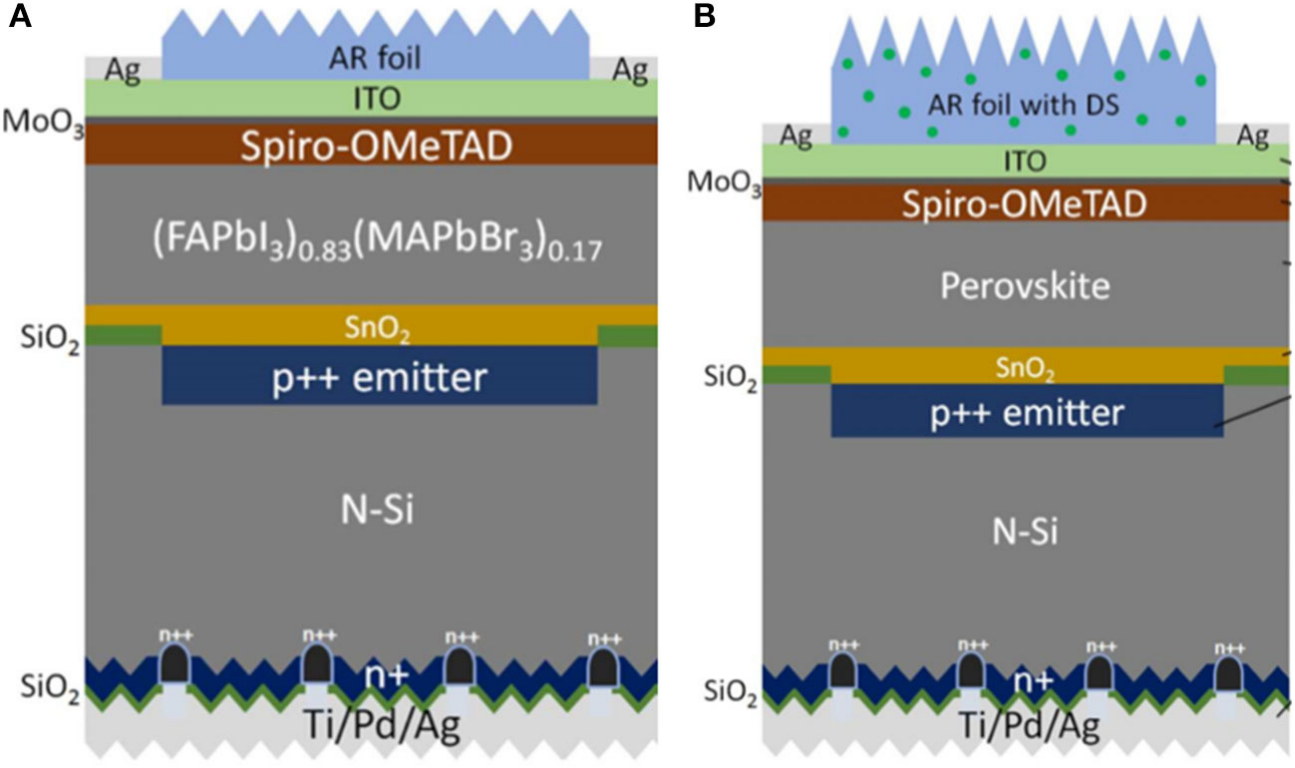
Schematic of PVSK/Si-homojunction solar **(A)** (reproduced from ref. 78, copyright 2018, The Royal Society of Chemistry) and **(B)** (reproduced from ref. 79, copyright 2018, ACS).

PERX Si have been used in the tandem cells for the high process compatibility. Wu et al. (2017) presented monolithic PVSK/Si tandem cells with a mesoscopic PVSK top subcell and PERT Si bottom subcell in 2017. With a metal stacking of Cr/Pd/Ag and optimal SiN_x_ thickness, they achieved an efficiency of 22.5% for the area of 1 cm^2^. In order to explore the possibility for tandem without introducing an extra recombination layer, Shen et al. (2018) fabricated 2 types of 2T tandem cells with homojunction PREC and SHJ employing carrier transport layer as a recombination layer achieving an efficiency of 22.9 and 24.1%, respectively, while the devices discussed above were fabricated at high temperature. Furthermore, Zheng et al. showed monolithic tandem cells with PVSK top subcell processed under a low temperature (<150°C) and PERT, PERC Si bottom subcell, respectively, for the first time, yielding a high efficiency of 17.6 and 21.8% for the large area of 16 cm^2^ as shown in Figure 11. In order to reduce parasitic absorption and improve the stability of devices, they (Zheng et al., 2019) applied textured polydimethylsiloxane (PDMS) films incorporating (Ba,Sr)_2_SiO_4_:Eu^2+^ micron phosphor on the tandem cells based on PERL Si bottom cell maintaining 90% of initial performance after 36 days of UV exposure.

#### IBC Cells

IBC is fabricated as the following process: n^+^ BSF is locally etching replaced by boron diffusion to form p^+^ emitter following with the deposition of SiO_2_ on the rear surface. The emitter and BSF are on the rear of IBC which might reduce sheet resistance, avoid shading of metal grid to achieve high J_SC_. Nevertheless, considering the rear-side lateral p-n junction of IBC Si could not match with the vertical p-n junction in PVSK, there was nearly no research about monolithic tandem cells replaced by 4T tandem cells.

For example, Jaysankar et al. (2018) adopted advanced light management by 3D-simulator incorporating the optimized thickness of each layer to achieve a refraction index matching with IBC Si bottom subcell. As a result, they yielded the efficiency of 23.9% on 4 cm^2^. Moreover, complex structural design of IBC limited the further utilization of the devices in tandem cells.

#### TOPCon Cells

Apart from the high efficiency that the PERX group and IBC have attained, complexly localized contact technology would lead to unavoidable carrier crowding at the opening and cause additional costs of Si. TOPCon adopt heavily doped poly-Si layer on a 2–3 nm rear tunnel SiO_x_ layer which could offer field-effect to help electrons achieve tunneling and block off holes avoiding local etching. As a result, this layer could attain the property of carrier selectivity. On the contrary, the tunnel layer and poly-Si layer could also be deposited on the front side of wafer with a rear Al_2_O_3_/SiN_x_ passivation layer.

**Figure 12:**
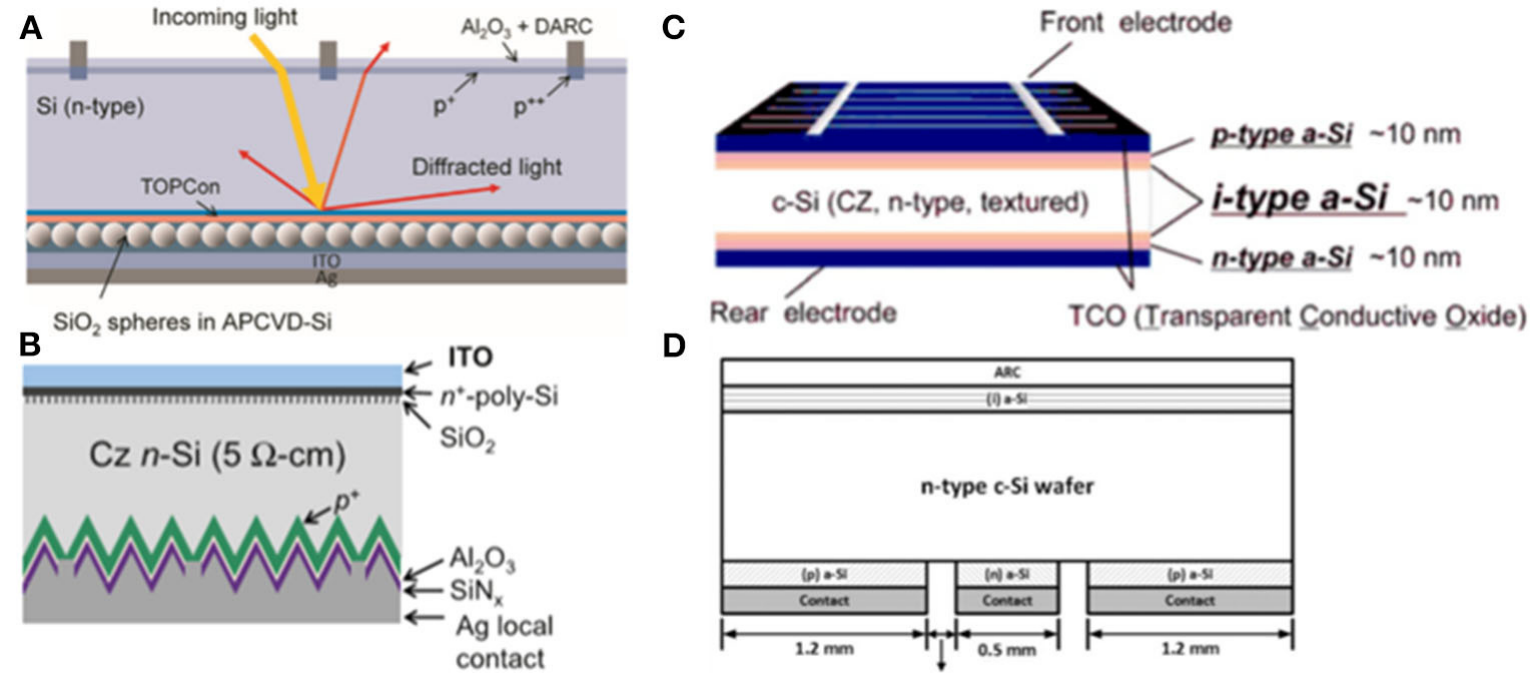
**(A)** TOPCon with rear side sphere gratings (reproduced from ref. 81, copyright 2018, Elsevier). **(B)** The cell structure with boron-diffused emitter on the textured side and tunnel oxide passivating nþ poly-Si contact on the other side (reproduced from ref. 14, copyright 2020, Elsevier). **(C)** Structure of an HITsolar cell. **(D)** Schematic of the HIT-IBC structure (reproduced from ref. 17, copyright 2015, Wiley VCH).

In 2015, Eisenlohr et al. constructed 2T tandem cells with TOPCon Si bottom subcell and PVSK top subcell adding diffractive structures of rear side hexagonal sphere gratings to enhance the light path length in the near infrared region in order to increase the absorption in Figure 12A (Eisenlohr et al., 2015). Based on this optical device, they estimated the efficiency could reach up to 22.4%. Nevertheless, damage would be caused with the following deposition process for TOPCon Si with a front side tunnel layer. Yoon et al. (2020) sputtered high transparency and low resistivity ITO recombination layer in PVSK/Si tandem cells demonstrating an approach to cure damage by annealing at 250°C in air as shown in Figure 12B. In contrast, Luderer et al. (2019) presented p^+^/n^+^ poly-Si as tunneling junction enabling low contact resistance and high V_OC_.

### Silicon Heterojunction Cells

Although homojunction Si possessed a series of merits including high temperature tolerance, large market share, and high efficiency, only a few tandem cells with homojunction Si as bottom cells were investigated for their limited voltage and lacked a recombination layer. On the contrary, SHJ with TCOs as a recombination layer could obtain a high V_OC_ exceeding 750 mV which is competitive for 2T tandem cells. Their emitter was formed by deposition and passivated with dielectric a-Si:H (i) without restriction of carrier transport. Moreover, the application of SHJ was broadened for a different refraction index, absorptivity, dielectric constant, and bandgap of two types of semiconductor materials in SHJ. They included a new configuration of heterostructure with intrinsic thin-layer (HIT) and further extended to HIT with interdigitated back contact (HIT-IBC), as shown in Figures 12C,D. Nevertheless, SHJ have a low temperature tolerance (<250°C) which is limited by the poor thermal stability of intrinsic a-Si:H layer. As a result, the PVSK top subcell should be fabricated at a low temperature.

HIT Si was constructed as follows: the a-Si:H (i) passivation layer and the a-Si:H (p) emitter layer was deposited on the front of the n-type wafer with the a-Si:H (i) layer and a-Si:H (n) on the rear. Then, TCO was deposited on both sides of devices with metal grid as contact. In spite of a small market share, most of the tandem cells employ HIT as a bottom subcell because of the simple processing without introducing an extra recombination layer.

**Figure 13:**
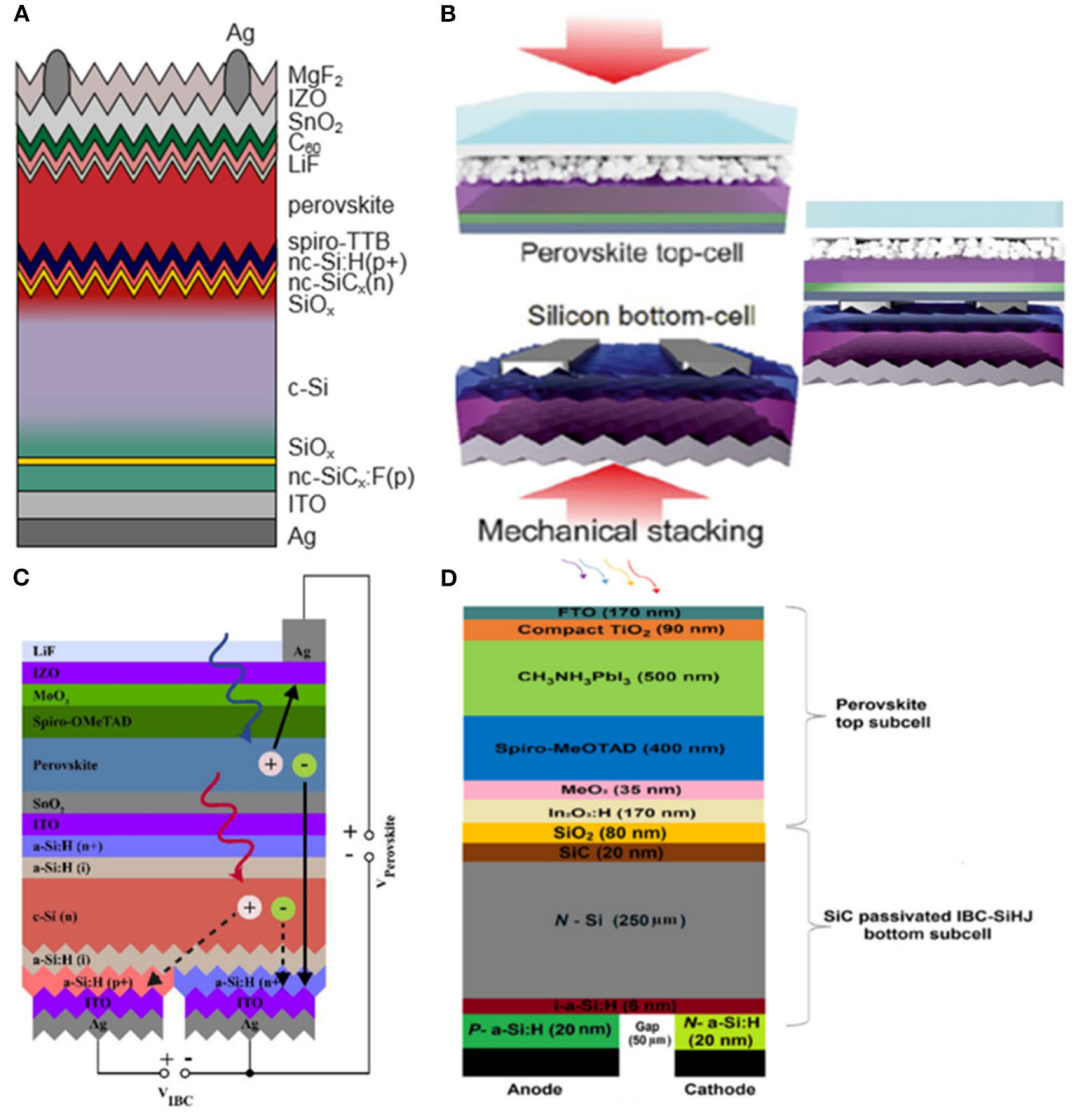
**(A)** Schematic view of the PVSK/p-type c-Si bottom cell (reproduced from ref. 85, copyright 2019, ACS). **(B)** Obtaining the 2T PVSK/Si tandem solar cell by mechanical stacking of subcells by applying pressure over their contact area (reproduced from ref. 86, copyright 2019, Elsevier Inc.). **(C)** Schematic drawing (not to scale) of IBC 3T tandem solar cell and its operating principle (reproduced from ref. 87, copyright 2020, ACS). **(D)** Device structures used in simulation of mechanically stacked PVSK/IBC-SiHJ tandem cells (reproduced from ref.88, copyright 2017, SPIE).

In 2016, Albrecht et al. demonstrated monolithic tandem cells with a SHJ Si bottom subcell (Albrecht et al., 2016b). They adopted a light management strategy with textured foil for anti-reflectance and light trapping enabling a high open circuit voltage of 1.78 V and efficiency of 18%. Considering about the regular p-i-n and invert n-i-p PVSK top cell, corresponding SHJ structure was divided into front emitter and rear emitter. Bush et al. (2017) showed an infrared-tuned Si heterojunction bottom subcell with rear emitter and inverted PVSK top subcell in tandem cells. They presented a bilayer of SnO_2_ and zinc tin oxide (ZTO) as a buffer layer to reduce parasitic absorption and prevent subsequent damage experiencing a 1,000-h test at 85°C and 85% humidity and obtaining the efficiency of 23.6%. On the contrary, Werner et al. (2016c) fabricated monolithic tandem cells with a front emitter heterojunction Si cell and p-i-n PVSK yielding efficiency up to 21.2% for the area of 0.17 cm^2^. In contrast with the n-type wafer discussed above, Nogay et al. (2019) demonstrated for the first time a p-type wafer Si subcell in tandem which was compatible with industrial standard Si cells obtaining a high efficiency of 25.1% as shown in Figure 13A. However, the low temperature tolerance of HIT restricted the use of mesoscopic PVSK in 2T tandem cells which could further improve efficiency. Lamanna et al. (2020) demonstrated 2T tandem cells by mechanically stacked PVSK top subcell and HIT Si bottom subcell which were fabricated independently and subsequently coupled back electrodes and front contact in Figure 13B. Furthermore, they introduced a graphene electron selective layer attaining the efficiency of 26.3% on the area of 1.43 cm^2^.

Replacing front TCO with SiN_x_ in HIT/IBC considering TCO on the front of the Si caused additional optical loss in the region of long wavelength could obtain both high V_OC_ from HIT structure and high J_SC_ from IBC. As a result, an up-to-date record of 26.6% had been reached for HIT/IBC.

The first experimental 3-teminal (3T) tandem cells with HIT/IBC Si subcell was demonstrated by Tockhorn et al. (2020) with an efficiency of 17.3%. Besides, they found a slight mutual dependence of two subcells from the resistance of electron contact shared by them in Figure 13C. Pandey and Chaujar (2017) designed 4T tandem cells with HIT/IBC bottom subcell in 2017 by a technology computer aided design (TCAD) device simulator as shown in Figure 13D. They showed the scope of the counter electrode's work function for extraction of holes by calculation achieving devices with an efficiency of 29.5%. Although there was high potential in efficiency for HIT/IBC, only very few tandem cells with them were presented for their complicated processing.

In summary, the monolithic tandem cells have achieved about an absolute 5% increase in efficiency compared with single-junction cells used no matter with homojunction or heterojunction Si. It indicates the generality of PVSK/Si solar cells which is feasible for almost all commercially available c-Si PV techniques. Moreover, this improvement is economically attractive given the current c-Si PV is already approaching the PCE ceiling. In the viewpoint of the c-Si solar cells, the PVSK/Si tandem devices provide an alternative route that is incremental rather than disruptive.

## Cost Analysis

c-Si solar cells have occupied more than a 90% share of the PV market with a total of 480 GW power in the worldwide by the end of 2018. The PCE could be improved by PVSK/Si tandem cells. The commercialization of PVSK/Si tandem solar cells requires high outputs, high stability, and low costs. As a result, “Grid parity” is defined by the generation of electronic costs of PV devices which could be comparable with the electricity prices from conventional energy installation. This concept is a valued benchmark but depends on the completeness and accuracy of the calculation method. A computing method called levelized cost of energy (LCOE) is presented to value the economic feasibility and consider grid parity of an energy generation project. Nevertheless, the calculation of LCOE always changes with the different assumptions which could be correct by the sensitivity analysis.

Song et al. calculated the LCOE of PSCs with the cost of 5.82 b kW h^−1^ assumed in Wichita, Kansas in the 30 years lifetime. Besides, they (Song et al., 2018) evaluated the manufacturing costs of 28.7, 33.8, 42.3 $ m^−2^ and minimum sustainable price of 0.32, 0.34, and 0.37 $ ${\text{W}}_{\text{p}}^{-1}$ for the single PSCs, 2T PVSK tandem and 4T PVSK tandem cells, respectively. Li et al. (2018) constructed 4 modules of PVSK, c-Si, PVSK/PVSK and PVSK/c-Si in LCOE to assess the competitiveness of PSCs and achieved the LCOE of 4.34 and 4.22 US cents kWh^−1^ for PVSK and PVSK/Si tandem cells, respectively. Besides, PVSK/Si tandem cells possessed the lowest LCOE compared to single junction Si and PVSK solar cells for their high efficiency. In addition to LCOE, they firstly demonstrated the concept of the LCOE decrease rate to analyze effectiveness regarding the research direction. In addition to the essential LCOE, the method to reduce costs according to manufacturing costs estimate as well as the uncertainty analysis was carried out by Chang et al. (2017). Except for above-mentioned analysis, energy yields were assessed in different locations with distinct climates: dry, temperate, and humid environment (Sofia et al., 2017). Besides, the calculations were further refined by residential district, commercial district and utility district (Fthenakis et al., 2017; Kaizuka et al., 2017). Nevertheless, there are some difficulties for devices with the lowest LCOE of PVSK and tandem cells possessing capital advantages to achieve industrialization for their restricted lifetimes. The lifetimes of 30 years are compulsively required for PV market access threshold without considering replacing disabled equipment before 30 years. Jean et al. (2019) proposed the strategy of periodical module replacement to resolve the disadvantages of restricted lifetimes for new-type PV devices. Furthermore, they showed the treatments of replacement materials by calculating and analyzing the influence of environment. This analysis provided another direction to think about the lifetime of devices.

Other different Si/PVSK tandem cells have also been analyzed to evaluate the possibility of market access. Li et al. (2018) constructed 2T PVSK/Si tandem cells with HIT Si cell achieving the LCOE of 5.22 US cents kWh^−1^ which is lower than single-junction PERC Si solar cell of 5.50 US cents kWh^−1^ but higher than single-junction PVSK solar cell of 4.34 US cents kWh^−1^ showing the competitiveness of tandem in the PV market. In addition, location of devices also has an influence on LCOE. Vernon et al. demonstrated the price for PVSK/SHJ tandem cells in the different residential energy production of Germany residential, and its range from 4.9 to 9 US cents kWh^−1^, which is competitive with wind, coal and gas. The low LCOE of tandem cells encourage further efforts to put tandem modules into the market in the future.

## Conclusion and Outlook

Looking back to the development of monolithic PVSK/Si tandem solar cells in recent years, the PCE was improved quickly from 13.7% to over 27% of 1 cm^2^. In the current design of the bottom cell, not only the passivation techniques have been applied, also the double-side textured wafer Si was successfully combined with top PVSK cells by solution processing. The structural evolution results in the device with a high EQE photocurrent and FF. However, the corresponding parameter in the bottom cell applied in the PVSK/Si tandem cells is far below that of the single junction c-Si cells with high performance. Therefore, there is still a large room for improving tandem solar cells based on bottom cells.

Compositional tuning of the top PVSK layer has been achieved in order to get a wide-band gap and good photo-stability device. Mixing the cations such as MA, FA, and Cs at A site; lead and tin at B site; and iodine, bromine, and chlorine in X site. Photo-induced phase segregation has been reduced and the material quality of the wide-band gap has been improved. Current matching between the top-cell and the bottom-cell is an important requirement for monolithically-integrated tandem solar cells because the photocurrent is determined by the smaller photocurrent of the two subcells, any current mismatch will lead to a large power loss. The sheet resistances of transparent electrode, ETL, and HTL could play an important role in determining the total PCE. As discussed in the previous section, reducing the thickness or the carrier concentration of transparent electrodes can suppress the parasitic absorption.

Future work could be focused on translating these proof-of-concept designs to scalable deposition processes, and many kinds of Si cells could be appropriate candidates owing to their specific advantages, including traditional c-Si, PERX, TOPCon, and SHJ. To be emphasized, TOPCon and SHJ, acting as bottom cells, are the main options for researchers to obtain tandem devices with high performance, because both of them demand special requirements on passivation quality. Especially TOPCon, with the establishment of a mature production line, could be another appropriate bottom cell for large-scale production. However, PERX family cells are worthy of investigation due to their predominant market share, which indicates strong motivation for the producers for further improvement by coupling with perovskite cells. The way of the tandem device commercialization is still hard, further advancement to resolve challenges related to PVSK stability is also undergoing extensive investigation. Encapsulation of tandem solar cells especially requires significant research. Once this has been accomplished, PVSK/Si tandems will be poised to be commercialized rapidly, benefiting from a partnership with the existing c-Si industry. Furthermore, more strategies for preparing large-area perovskite solar cells should be explored, while most research is focusing on small-area exploration in the laboratory. Standards for testing stability are needed, and the corresponding studies should report stability data exhaustively. We believe that the commercialization of PVSK/Si tandem, which provides one of the most promising approaches to producing cheaper solar electricity, can be achieved in the near future.

## Author Contributions

All authors listed have made a substantial, direct and intellectual contribution to the work, and approved it for publication.

## Conflict of Interest

The authors declare that the research was conducted in the absence of any commercial or financial relationships that could be construed as a potential conflict of interest.
